# A Critical Role of Culture Medium Selection in Maximizing the Purity and Expansion of Natural Killer Cells

**DOI:** 10.3390/cells13131148

**Published:** 2024-07-05

**Authors:** Neele Kusch, Jonathan Storm, Antonia Macioszek, Ella Kisselmann, Cornelius Knabbe, Barbara Kaltschmidt, Christian Kaltschmidt

**Affiliations:** 1 Department of Cell Biology, Bielefeld University, 33615 Bielefeld, Germany; jonathan.storm@uni-bielefeld.de (J.S.); antonia.macioszek@gmx.de (A.M.); ella.kisselmann@uni-bielefeld.de (E.K.); barbara.kaltschmidt@uni-bielefeld.de (B.K.); c.kaltschmidt@uni-bielefeld.de (C.K.); 2 Forschungsverbund BioMedizin Bielefeld/OWL FBMB e.V., 33617 Bielefeld, Germany; cknabbe@hdz-nrw.de; 3 Institute for Laboratory and Transfusion Medicine, Heart and Diabetes Centre NRW, Ruhr-University Bochum, 32545 Bad Oeynhausen, Germany; 4 Medical Faculty Ostwestfalen-Lippe, University of Bielefeld, 33615 Bielefeld, Germany; 5 Molecular Neurobiology, Bielefeld University, 33615 Bielefeld, Germany

**Keywords:** natural killer cells, adherent selection, serum, medium, NK MACS medium, common gamma chain cytokines, activation beads, cytotoxicity, NK cell receptors, flow cytometry

## Abstract

Natural killer (NK) cells hold promise in cancer treatment due to their ability to spontaneously lyse cancer cells. For clinical use, high quantities of pure, functional NK cells are necessary. Combining adherence-based isolation with specialized media showed the unreliability of the isolation method, but demonstrated the superiority of the NK MACS^®^ medium, particularly in suboptimal conditions. Neither human pooled serum, fetal calf serum (FCS), human platelet lysate, nor chemically defined serum replacement could substitute human AB serum. Interleukin (IL-)2, IL-15, IL-21, and combined CD2/NKp46 stimulation were assessed. IL-21 and CD2/NKp46 stimulation increased cytotoxicity, but reduced NK cell proliferation. IL-15 stimulation alone achieved the highest proliferation, but the more affordable IL-2 performed similarly. The RosetteSep™ human NK cell enrichment kit was effective for isolation, but the presence of peripheral blood mononuclear cells (PBMCs) in the culture enhanced NK cell proliferation, despite similar expression levels of CD16, NKp46, NKG2D, and ICAM-1. In line with this, purified NK cells cultured in NK MACS^®^ medium with human AB serum and IL-2 demonstrated high cytotoxicity against primary glioblastoma stem cells.

## 1. Introduction

Kiessling et al. and Herberman et al. initially identified NK cells in a mouse model as a unique subset of lymphocytes, capable of independently killing tumor cells without prior sensitization [1,2,3,4]. NK cells are a part of the innate immune system and crucially contribute to host defense against stressed virally transformed cells, and tumor development [5]. Phenotypically, NK cells are identified by their expression of the surface protein CD56, also known as the neural cell adhesion molecule (NCAM), and lack of CD3 expression [6]. In humans, NK cell subsets are classified based on CD56 surface density (CD56^dim^ or CD56^bright^), along with the presence or absence of CD16 [7]. While CD56^bright^ cells are primarily regarded as cytokine producers and CD56^dim^ cells as cytotoxic, both subtypes can engage in both functions. Notably, only CD56^dim^ cells participate in antibody-dependent cellular cytotoxicity (ADCC), facilitated by their high CD16 expression [8,9,10,11]. NK cells integrate signals from a plethora of activating and inhibitory receptors. Therefore, abnormal cells are either identified by the detection of a lack of inhibitory molecules like major histocompatibility complex (MHC) class I or an abundance of activating ligands such as stress-induced MICA [12,13,14]. NK cells express various activating receptors including natural cytotoxicity receptors NKp30, NKp44, and NKp46, as well as the accessory molecule DNAX (DNAM-1) and natural killer group 2D (NKG2D) [5,15,16]. NK cells also express adhesion molecules for migration and homing. Notably, they express the co-stimulatory receptor lymphocyte function-associated antigen-1 (LFA-1), which recognizes intercellular adhesion molecule-1 (ICAM-1), but they also upregulate the expression of ICAM-1 itself after stimulation, providing a stimulatory link to the adaptive immune system [17].

The potent innate antitumor activity of NK cells against cells with oncogenic transformation markers, coupled with their limited reactivity to healthy tissue, makes them promising candidates for cancer therapy [18,19]. Clinical use of NK cells has advanced significantly, partly due to the high cancer-related morbidity caused by aggressive treatments like chemotherapy and radiation. Although these treatments eliminate rapidly proliferating tumor cells, they are less effective against cancer stem cells, which cause relapses [20,21]. Unlike chemotherapy and radiation, immunotherapy does not rely on cancer cell proliferation. Additionally, the epithelial-mesenchymal transition, a mostly dedifferentiating process occurring in cancer progression, induces NKG2D ligand expression, which subsequently activates NK cells [22,23]. The resulting ability to target cancer stem cells strongly supports the use of NK cell-based therapies for combating cancer [24,25]. Due to their increased cytotoxicity and minimal toxicities toward recipients, infusion of ex vivo activated NK cells as adoptive cell therapy is an effective approach [9]. However, adequate NK cell infusions are necessary to achieve the right effector-to-target ratio. Since the most common NK cell source, peripheral blood mononuclear cells (PBMCs), only contains a small fraction. Thus, expansion is essential to generate sufficient fully functional NK cells for this approach [9,26,27]. The ability of NK cells to distinguish between healthy and transformed or infected cells through germ-line encoded receptors makes them ideal for allogeneic applications. Since PBMCs contain a substantial number of T cells that could potentially cause graft-versus-host disease (GvHD), purification may be needed to achieve sufficient NK cell purity [28].

NK cells are often isolated using magnetic cell sorting with antibodies conjugated to magnetic beads [29]. However, the expensive materials for selection lead to a significant cost factor. Adherence-based selection is supposed to be a more affordable alternative, which begins with cultivating PBMCs and achieves higher NK cell purity during cultivation. In this method, PBMCs are stimulated with interleukin-2 (IL-2) and ionomycin, which are known to induce activation and adhesion of NK cells, allowing selection by removing non-adherent cells [30]. Typically, a primary mitogenic stimulus and cell-cell contact-dependent costimulatory signals are needed for optimal primary NK cell proliferation [30,31,32]. Ionomycin is a calcium ionophore, routinely used with phorbol 12-myristate 13-acetate (PMA) to achieve non-specific lymphocyte activation, inducing cytokine production and degranulation, which both require calcium ions [33,34,35]. In this protocol, IL-2 serves as the primary mitogenic stimulus, while ionomycin provides essential early costimulatory signals. The calcium ion channel-opening antibiotic simulates activating receptor signaling, bypassing the need for cell-cell contacts [36,37,38].

Many different culture media have been applied in NK cell culture so far, ranging from standard RPMI-1640 to DMEM and DMEM/F12-based formulations [39], more specialized formulations for hematopoietic cells like CellGenix^®^ SCGM, Lonza™ X-VIVO™ 10, and Gibco AIM V™ [40], as well as media specifically formulated for NK cells like Miltenyi Biotec NK MACS^®^ [41] and FUJIFILM Irvine Scientific PRIME-XV NK cell CDM [42]. NK cell culture medium is typically supplemented with either fetal calf serum (FCS) or human serum, and the application of human platelet lysate and serum replacements has been reported [39,41,43,44,45]. Notably, PRIME-XV NK cell CDM is the first commercially available animal component-free chemically defined medium specifically designed for NK cell culture.

Previous studies have shown that the cytokines IL-2, IL-15, and IL-21 can modify the peripheral repertoire of NK cells [46]. These cytokines signal through the common gamma chain receptor family and enhance cellular survival and proliferation by activating the following pathways: phosphatidylinositol 3-kinase (PI3-K), Akt, RAS/mitogen-activated protein kinase (MAPK), and Janus-associated kinases (JAK)/signal transducers and activators of transcription (STAT) [47,48]. IL-2, the most common interleukin, is relatively inexpensive and boosts NK cell cytotoxicity [49]. IL-15 synergistically increases NK cell numbers [46]. The addition of IL-21 enhances NK cell cytotoxicity by stimulating interferon-ɣ production, demonstrating strong antitumor activity in clinical trials [49,50]. While IL-21 alone does not induce NK cell proliferation [46,51], it is beneficial for optimal cytotoxic function [50,52].

Taken together, the expansion of NK cells can be costly and challenging, with many protocols failing to yield sufficient cell numbers for successful therapy. Multiple factors influence the expansion and activation of NK cells, making optimization of cultivation components essential for developing a feasible, cost-effective clinical expansion method. These components include the choice of purification protocols, culture media, sera, cytokines, and specialized stimuli. We initially hypothesized that modifying the adherence-based selection with specialized NK cell culture media could provide a cost-effective method for NK cell enrichment with high proliferation rates. Although we encountered several limitations of the adherence-based selection method, the chosen medium played a critical role in NK cell culture, particularly in suboptimal conditions. Therefore, we investigated how various cultivation factors impact the proliferation and activation state of NK cells in the selected medium. Specifically, we compared different sera or serum replacements, cytokines, and the impact of stimulating beads on NK cell proliferation, purity, and cytotoxicity. Additionally, we tracked activating receptor expression on NK cell surfaces during cultivation and examined how the presence of other PBMCs influences their expression.

## 2. Materials and Methods

### 2.1. Isolation of PBMCs and NK Cells

PBMCs and NK cells were isolated from buffy coats of healthy donors, kindly provided by the Institut für Laboratoriums und Transfusionsmedizin, HDZ NRW, Bad Oeynhausen, Germany. Healthy donors were selected according to the German Hemotherapy Guidelines. The buffy coats were produced from blood donations during routine processing. The collected whole blood was centrifuged and the three distinct layers: red blood cells at the bottom, plasma at the top, and a thin middle layer known as the buffy coat were transferred to new bags by use of an expressor. The buffy coat contains a concentrated mixture of leukocytes and platelets but also some remaining plasma and red blood cells. The leukocytes, or more specifically PBMCs, were therefore isolated via density gradient centrifugation using Lymphoprep™ and SepMate™ tubes (both Stemcell Technologies Germany GmbH, Cologne, Germany). For the isolation of NK cells, the RosetteSep™ human NK cell enrichment kit (Stemcell Technologies Germany GmbH, Cologne, Germany) was used according to the manufacturer’s instructions.

### 2.2. Enrichment of NK Cells through Adherent Selection

#### 2.2.1. Seeding

To investigate how different media influence the adherent selection method, three different media were tested. PBMCs obtained from donors 5 to 8 were cultured in PRIME-XV NK Cell CDM (FUJIFILM Irvine Scientific, Tilburg, Netherlands) with the addition of 1% penicillin–streptomycin (Sigma-Aldrich GmbH, Steinheim, Germany) and Dulbecco’s Modified Eagle Medium: Nutrient Mixture F-12 (DMEM/F-12) (Sigma-Aldrich GmbH, Steinheim, Germany) supplemented with 10% allogenic human AB serum (lab-made [53]), 2 mM L-alanyl-l-glutamine (Sigma-Aldrich GmbH, Steinheim, Germany), and 1% penicillin–streptomycin (Sigma-Aldrich GmbH, Steinheim, Germany). NK MACS^®^ medium (Miltenyi Biotec GmbH, Gladbach, Germany) was applied to donors 9 to 12. NK MACS^®^ basal medium was supplemented with NK MACS^®^ supplement, 5% human AB serum (PAN Biotech™ GmbH, Aidenbach, Germany), and 1% penicillin–streptomycin (Sigma-Aldrich GmbH, Steinheim, Germany).

For the adherent selection of NK cells, 1 × 10^8^ cells were seeded in 20 mL of the respective medium (5 × 10^6^ cells/mL) in tissue culture plates (diameter 15 cm). Additionally, 0.75 µg/mL ionomycin (Santa Cruz Biotechnology, Heidelberg, Germany) and 1000 U/mL IL-2 (Miltenyi Biotec GmbH, Gladbach, Germany) were applied for stimulation. The cells were incubated overnight at 37 °C and 5% CO_2_. 

#### 2.2.2. Adherence-Based Selection

The adherent selection method starts with the cultivation of a heterogeneous population of cells, the isolated PBMCs, followed by the removal of the non-adherent cells. Accordingly, in the first step, the medium with the included non-adherent cells was removed from the culture dishes. The dishes were washed three times with warm PBS (Sigma-Aldrich GmbH, Steinheim, Germany) to remove residual non-adherent cells. The removed cells were put aside for further examination by flow cytometry. The remaining adherent cells were not analyzed to avoid disturbance at that early stage of culture. For cultivation at 37 °C and 5% CO_2_, 20 mL of the corresponding medium (PRIME-XV NK Cell CDM, DMEM/F-12 or NK MACS^®^ medium) and IL-2 at a concentration of 1000 U/mL were added to the remaining cells.

#### 2.2.3. Cultivation of the Adherently Selected Cells

Every two to three days, the cultures were qualitatively assessed for medium acidification as an indicator of cell metabolism (DMEM/F12 and NK MACS^®^). If noticeable discoloration of the culture medium to orange/yellow occurred, the cultures were fed with 10 mL of fresh medium and IL-2. If no discoloration was visible, the cultures were fed with 5 mL of fresh medium and IL-2. The amount of IL-2 was calculated as 500 U/mL of culture volume, including the fresh medium (e.g., 20 mL fed with 10 mL equals 30 mL and, therefore, 15,000 U IL-2; 30 mL fed with 5 mL equals 35 mL and, therefore, 17,500 U IL-2). The concentration of 500 U/mL is based on the protocol by Selvan and Dowling and also corresponds to the recommended IL-2 concentration for NK cell culture by Miltenyi Biotec [30,54]. The decision to feed was reassessed by visual inspection using an inverted phase contrast microscope. If higher cell density was observed despite no media discoloration, the cultures were still fed with 10 mL of fresh culture medium. For cultures in PRIME-XV NK Cell CDM, cell growth was only assessed via microscopy, as this medium does not contain the pH indicator phenol red. Once a feed would have required the culture volume to exceed 40 mL, the cells were either passaged or concentrated. The passage decision required either adherent cells at above 80% confluency or a floating cell density with barely any in-between spaces and difficulty in discriminating individual cells. For passage, the medium containing the floating cells was removed, and the adherent cells were washed with PBS and subsequently detached with trypsin EDTA solution (Sigma-Aldrich GmbH, Steinheim, Germany). The medium, PBS, and trypsin EDTA solution were pooled and centrifuged (10 min, 300 g, RT, brake 3). The cells were reseeded at a density of 0.5–1 × 10^6^ cells per ml in fresh culture medium with 500 U/mL IL-2 in a new tissue culture plate (in culture plates of 15 cm diameter with 20 mL medium or culture plates of 10 cm diameter with 8 mL medium, according to the determined cell count). For 15 cm plates, cultures were further fed according to the description above. For 10 cm plates, all volumes were scaled down by a factor of 2.5. For the concentration of cells, media was removed until 10 mL remained. The removed cells were recovered by centrifugation (10 min, 300 g, RT, brake 3) and added back to the plate with 10 mL fresh culture medium and 500 U/mL IL-2.

### 2.3. Isolation and Cultivation of RosetteSep™ Human NK Cell Enrichment Kit Purified NK Cells

As the adherent selection of PBMCs showed strong variability and resulted in insufficient expansion, the supplements previously identified as optimal for NK cell culture were retested on PBMCs and isolated NK cells (see Section 2.1). This time, in an additional approach, the buffy coat was concentrated beforehand. For this purpose, the buffy coats mentioned in Section 2.1 were centrifuged (30 min, 800 g, RT, break off) to achieve separation between the cells and the plasma. Subsequently, half of the total volume was removed from the upper fraction consisting of plasma. This resulted in a two-fold concentrated buffy coat. To refer to normal, not concentrated buffy coats, the terminology “untreated buffy coat” is used in the following. Consequently, in this approach, NK cells were isolated under the application of the RosetteSep™ human NK cell enrichment kit out of untreated and two-fold concentrated buffy coats. Subsequently, 2 × 10^6^ cells were seeded in NK MACS^®^ basal medium supplemented with NK MACS^®^ supplement (Miltenyi Biotec GmbH, Gladbach, Germany), 1% penicillin–streptomycin (Sigma-Aldrich GmbH, Steinheim, Germany), 5% human AB serum (PAN Biotech™ GmbH, Aidenbach, Germany) and 500 U/mL IL-2 (Miltenyi Biotec GmbH, Gladbach, Germany) and cultured at 37 °C, 5% CO_2_ in an incubator (Binder, Tuttlingen, Germany). The cells were fed with fresh medium and IL-2 twice a week and were passaged once a week.

### 2.4. Culture of NK Cells for the Proliferation Assays

Isolated NK cells were cultivated in NK MACS^®^ basal medium with the addition of the accompanying NK MACS^®^ supplement (Miltenyi Biotec GmbH, Gladbach, Germany), 1% penicillin–streptomycin (Sigma-Aldrich GmbH, Steinheim, Germany), 5% of the corresponding serum or serum replacement and the appropriate cytokine. The cells were seeded in a 12-well plate (Sarstedt AG & Co., Nümbrecht, Germany) and cultured at 37 °C, 5% CO_2_ in an incubator (Binder, Tuttlingen, Germany). The medium and cytokines were changed every week by passaging the cells using trypsin EDTA solution (Sigma-Aldrich GmbH, Steinheim, Germany) to detach the adherent cell portion. Additionally, the culture volume was doubled through the addition of fresh medium once a week. Moreover, cytokines were replenished to the corresponding concentration for the total volume. If the cell count exceeded 1 × 10^6^, a part of the cells was removed from the experiment. In general, the following cytokines were used: IL-2, IL-15, and IL-21.

### 2.5. Serum and Proliferation Assay

For the proliferation assay FCS (Thermo Fisher Scientific Life Technologies, Darmstadt, Germany), human AB serum (lab-made [53]), human pooled serum (lab-made [53]), MultiPL’100 human platelet lysate (Macopharma, Mouvaux, France), and Panexin CD (PAN Biotech™, Aidenbach, Germany) were applied. The cells were seeded with a cell density of 4 × 10^5^ per ml. For cytokine stimulation, 500 U/mL IL-2 (PeproTech, Hamburg, Germany/Miltenyi Biotec GmbH, Gladbach, Germany) were supplemented. Three assays were performed: The first with NK cells isolated from donor 1, the second with NK cells from donor 1 mixed with those from donor 2, and the last with NK cells isolated from donor 3. All following assays were performed with NK MACS^®^ basal medium supplemented with NK MACS^®^ supplement (Miltenyi Biotec GmbH, Gladbach, Germany), 1% penicillin–streptomycin (Sigma-Aldrich GmbH, Steinheim, Germany), and 5% human AB serum (PAN Biotech™ GmbH, Aidenbach, Germany).

### 2.6. Long-Term Proliferation Assay with NK Cell Activation/Expansion Beads

NK cells obtained from donor 3 were seeded with a cell density of 4 × 10^5^ per mL. The anti-biotin MACSiBead™ particles from the NK cell activation/expansion kit (Miltenyi Biotec GmbH, Gladbach, Germany) were prepared and used according to the manufacturer’s instructions (one bead per two cells). The NK cells were cultured under four different conditions for five weeks: first, with NK cell activation/expansion beads and IL-2 (500 U/mL) (Miltenyi Biotec GmbH, Gladbach, Germany), second, with activation/expansion beads, third, IL-2 (500 U/mL) only or fourth, without any stimulatory factors (Miltenyi Biotec GmbH, Gladbach, Germany). 

### 2.7. Short-Term Proliferation Assay with NK Cell Activation/Expansion Beads and Cytokines

Isolated NK cells from donor 4 were seeded with a cell density of 1 × 10^6^ per mL. For stimulation, the cytokines IL-2 (500 U/mL) (Miltenyi Biotec GmbH, Gladbach, Germany), IL-15 (100 U/mL) (Miltenyi Biotec GmbH, Gladbach, Germany) and IL-21 (1 U/mL) (Miltenyi Biotec GmbH, Gladbach, Germany) were used in different combinations with and without NK cell activation/expansion beads (Miltenyi Biotec GmbH, Gladbach, Germany). The cells were cultured for 10 days and fed with fresh medium and cytokines on days 3, 5, and 7. Additionally, on day 5, the cells were passaged into a 6-well plate. 

### 2.8. Evaluation of the Cytotoxicity of the Stimulated NK Cells

K562 cells (ATCC^®^) were stained with 2.5 µM CellTrace™ Violet (Life Technologies, Eugene, Oregon) diluted in PBS (Sigma-Aldrich GmbH, Steinheim, Germany). For the killing assays, these cells were then incubated with the stimulated NK cells in a 1:1 ratio (10^5^ cells) in RPMI 1640 medium containing 1% penicillin–streptomycin (Sigma-Aldrich GmbH, Steinheim, Germany) and 10% FCS (Thermo Fisher Scientific Life Technologies, Darmstadt, Germany) in a 12-well plate at 37 °C, 5% CO_2_. After an incubation time of four hours, the cells were harvested, washed with PBS, and incubated for 30 min at 2–8 °C in 1:1000 GloCell™ Fixable Viability Dye Red 710 (Stemcell Technologies GmbH, Köln, Germany) in PBS. Subsequently, the cells were washed with PBS containing 2% FCS and the pellets were resuspended in 0.1% Paraformaldehyde (VWR International GmbH, Hannover, Germany) in PBS and stored at 2–8 °C until analysis by flow cytometry on the next day.

(1) $$\mathrm{Specific}\;\mathrm{lysis}\;[\%]=\frac{living\;cells\;control\;[\%]-living\;cells\;sample\;[\%]}{living\;cells\;control\;[\%]}\;\times \;100$$

Equation (1). Calculation of the specific lysis with the percentages determined via flow cytometry in the cytotoxicity assay.

### 2.9. NK Cell Activating Receptor Analysis

Freshly isolated PBMCs and NK cells obtained from 3 different donors were seeded in 6-well plates (Sarstedt AG & Co., Nümbrecht, Germany) with 2 × 10^6^ cells per well in 2 mL NK MACS^®^ medium with IL-2 Proleukin S (Clinigen Healthcare Ltd., Weybridge, UK) (500 U/mL) and cultured at 37 °C, 5% CO_2_ in an incubator (Binder, Tuttlingen, Germany). The cells were either passaged or fed with fresh medium and IL-2 every two to three days during the three weeks of culture. The cell analysis of the cell number and NK cell receptor expression took place after isolation and on days 4, 7, 12, 15, 18, and 21.

### 2.10. Quantification of Cells

A counting chamber with a depth of 0.1 mm (Brand GmbH, Wertheim, Germany) was used to determine the cell numbers. The cells were stained with Trypan Blue Solution (1:2) (Sigma-Aldrich Chemie GmbH, Steinheim, Germany) prior to counting, in order to selectively color dead cells. If the concentration of cells was too high, they were additionally diluted with PBS (Sigma-Aldrich GmbH, Steinheim, Germany). In the adherence-based selection method, the total cell expansion was calculated under the assumption that the initially seeded cell amount minus the removed cells equals the starting cell number. The numbers of NK cells were determined as the product of the total cell number with the NK cell portion determined by flow cytometry. All expansion rates and cell numbers represent theoretical values as if no cells were removed during culture.

### 2.11. Flow Cytometric Analysis

Flow cytometric measurements were performed with a Gallios™ Cell Analyzer—10 Colors, 3 Lasers (Beckman Coulter Life Sciences, Krefeld, Germany).

#### 2.11.1. Assessment of NK Cell Purity

For identification of NK, T as well as NKT cells, the cells were stained with 4′,6-Diamidino-2-phenylindol (DAPI, Sigma-Aldrich) and CD56-APC-A700 (Beckman Coulter Inc., Brea, CA, USA), as well as CD3-FITC (BioLegend, San Diego, CA, USA) antibodies (Table 1). The gates were set to selectively contain events in a stable flow (Figure S1). Doublets and debris were removed by applying scatter plots. Moreover, dead cells were excluded by setting the gates to exclude DAPI-stained cells. The percentage of detected NK (CD3^−^CD56^+^), T (CD3^+^CD56^−^), or NKT (CD3^+^CD56^+^) cells in the living cells was multiplied by the percentage of detected lymphocytes.

Actual NK, T, or NKT cell fraction [%] = (lymphocytes [%] × identified cell population [%]) × 100(2)

Equation (2). Calculation of the actual percentages of NK, T, and NKT cells in freshly isolated or cultivated cells.

#### 2.11.2. Assessment of NK Cell Receptor Expression

To identify NK cells and assess the expression levels of activating NK cell receptors, the cells were stained with seven different fluorochrome-labeled antibodies, in the presence of 10% human tandem signal enhancer (Miltenyi Biotec, Bergisch Gladbach, Germany) and a viability dye (Table 2). Gates were set using fluorescence minus one controls with a false positive threshold of a maximum of 0.5%. The gating strategy was set to select events in stable flow, singlets, cells, and living CD45^+^ lymphocytes before determining CD3^−^ cells (Figure S2). NK cell frequency was calculated as the product of the frequency of CD56^+^ cells in CD3^−^ cells with the frequency of CD3^−^ cells in CD45^+^ cells and the frequency of CD45^+^ cells in DAPI^−^ cells, as the lymphocytes in this case could be identified by CD45 expression. The activating receptor-positive cell percentage is always mentioned as the content of NK cells.

Actual NK cell fraction [%] = (CD45^+^ [%] × CD3^−^ [%] × CD56^+^ [%]) × 100 (3)

Equation (3). Calculation of the actual percentages of NK cells in freshly isolated or cultivated cells using the 8-color flow cytometry panel.

### 2.12. NK Cell Cytotoxicity Capacity against Glioblastoma Cancer Stem Cells

For the assessment of glioblastoma cancer stem cell lysis, RosetteSep™ purified NK cells from a human buffy coat were cultured in NK MACS^®^ medium supplemented with human AB serum and 500 U/mL IL-2 (Miltenyi Biotec, Bergisch Gladbach, Germany). At days 13, 20, and 26 NK cells were applied in killing assays against primary glioblastoma cancer stem cells (which were characterized in [55]). The assay was conducted according to the protocol described in Section 2.8.

### 2.13. Analysis and Illustration

After conducting flow cytometric measurements using Kaluza for Gallios™ software (version 1.2), the Kaluza for Analysis software (version 1.2) (Beckman Coulter Life Sciences, Krefeld, Germany) was used for the identification of NK cells (Section 2.11.1), while the receptor expression was analyzed via the CellEngine^®^ software (CellCarta, Montreal, QC, Canada) (Section 2.11.2). For the analysis of the cell proliferation, we used Excel version 2211 (Microsoft, Redmond, WA, USA). For the visualization of the data and statistical tests, we used GraphPad Prism version 8.3.0 (538) (GraphPad Software, Boston, MA, USA). Schematic illustrations were created with BioRender.com (accessed on 29 May 2024).

## 3. Results

### 3.1. Adherent Selection Is Strongly Influenced by the Culture Medium, Is Highly Variable, and Leads to Low NK Cell Expansion

**Figure 1:**
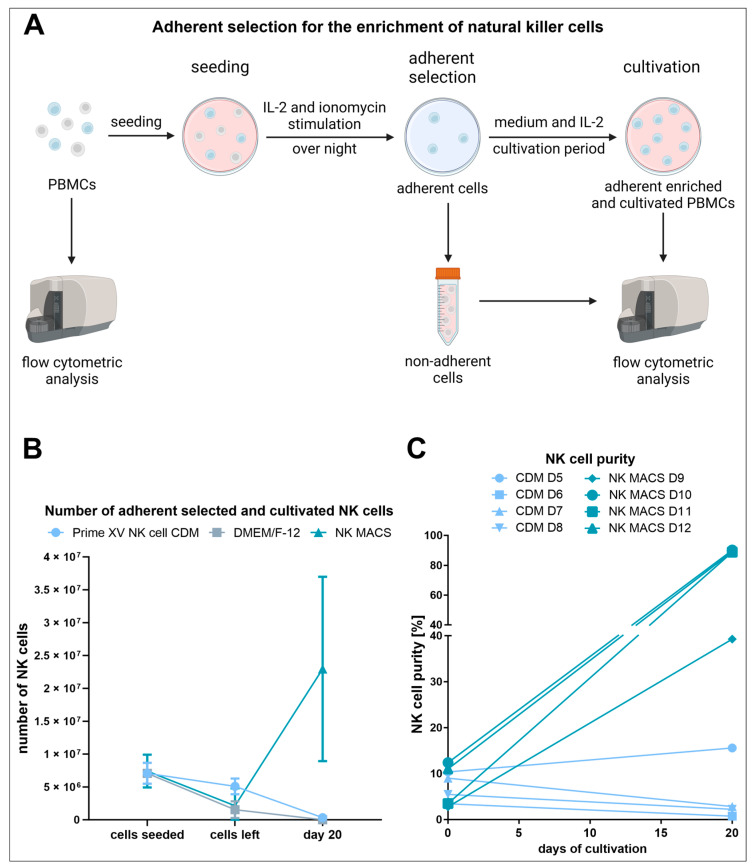
Freshly isolated PBMCs were cultured in different media and adherently selected for NK cell enrichment. (**A**) Schematic illustration of the adherent selection process for the enrichment of NK cells: the culture begins with PBMCs, which are activated overnight to enable the selection of adherent cells on the next day. Then, the adherent cells are further cultured. Flow cytometry analysis was performed with the PBMCs before seeding, the cells that were removed during the adherent selection process, and the adherently selected and cultivated cells. (**B**) The number of NK cells left after the adherent selection process. (**C**) The percentage of CD56^+^/CD3^−^ NK cells in freshly isolated PBMCs and achieved purity of adherently selected cell cultures of different donors after culture in PRIME-XV NK Cell CDM (CDM) or NK MACS^®^ (NK MACS), as determined by flow cytometry.

Although the scale would not allow parallel comparisons of the same donors in the three tested media, the adherent selection was performed on the large scale used by Selvan and Dowling, with the aim of achieving a large quantity of high-purity NK cells within a certain cultivation period [30]. To obtain NK cells by adherent selection, PBMCs were isolated from buffy coats of healthy donors and cultured in either PRIME-XV NK Cell CDM, DMEM/F-12, or NK MACS^®^ medium. As for these large-scale approaches, no direct comparison for all three media was possible due to constraints in cell numbers, other donors were used in the NK MACS^®^ medium. After overnight stimulation of the PBMCs with IL-2 and ionomycin, the non-adherent cells were removed, and the adherent cells were further cultured (Figure 1A). Microscopic images were used to monitor cell density and morphology during these steps (Figure S3). After overnight stimulation of PBMCs with IL-2 and ionomycin, the non-adherent cells were removed, and the adherent cells were further cultured (Figure 1A). Cell morphology and density were media-dependent after non-adherent cell removal and during culture (Figure S3). Surprisingly, PRIME-XV NK Cell CDM led to a much less pronounced total cell reduction through the removal of the non-adherent cells than DMEM/F12 and NK MACS^®^, both with similar numbers (Figure S5B, Table 3 and Table S1). Similarly, only PRIME-XV NK Cell CDM showed lower NK cell contents in the removed cells when compared to the initial PBMC population, resulting in a less pronounced removal of NK cell numbers (Figure 1B, Figures S5A and S6B, Table 3 and Table S1). Total cell numbers continued to decline during culture (Figures S5B and S6A). In essence, only culture in NK MACS^®^ resulted in NK cell expansion (Figure 1B, Figures S5C and S6D, Table 3 and Table S1). This was also reflected in the NK cell purity, reaching a mean of 89% (Figure 1C and Figure S6C, Table 3 and Table S1). Cryopreserved cells of a donor previously used with PRIME-XV NK Cell CDM and DMEM/F12 yielded roughly 86% pure NK cells under the use of NK MACS^®^ (Figure S7).

### 3.2. NK Cell Isolation via the RosetteSep™ Human NK Cell Enrichment Kit Yielded High Purities but Decreased NK Cell Expansion Due to the Absence of Other Stimulating PBMCs

**Figure 2:**
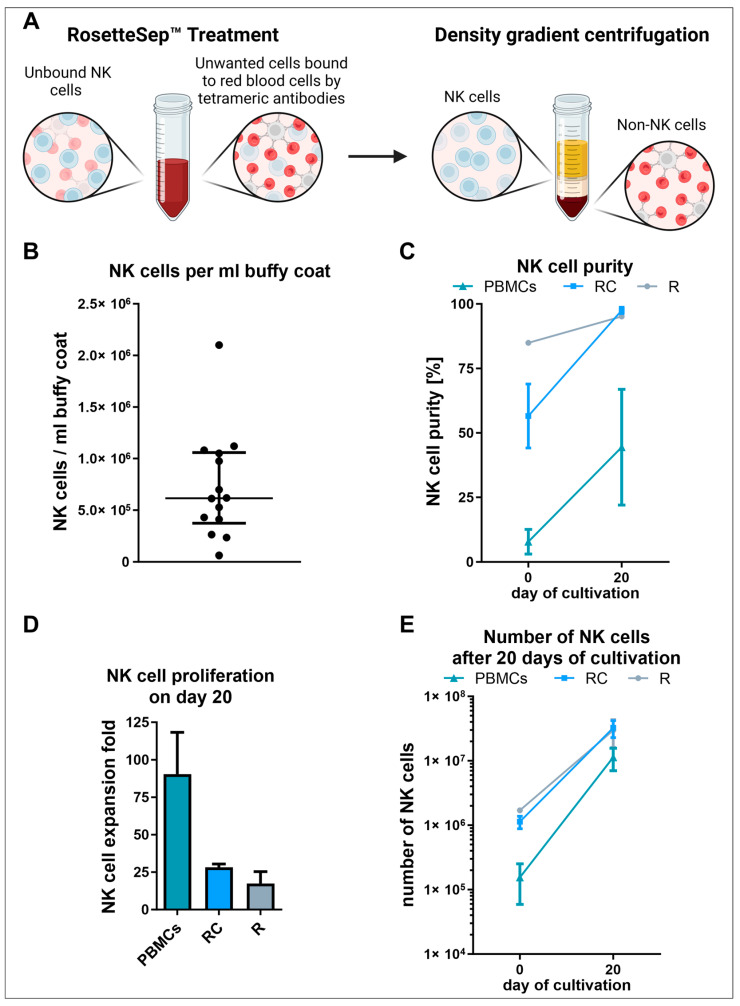
Freshly isolated PBMCs and RosetteSep™ human NK cell enrichment kit purified NK cells were cultured in NK MACS^®^ medium supplemented with human AB serum and 500 U/mL IL-2 for 20 days. For the isolation of NK cells, untreated (R) and two-fold concentrated (RC) buffy coats were used. (**A**) Schematic representation illustrating the NK cell purification process utilizing the RosetteSep™ cocktail. The cocktail comprises tetrameric antibodies that bind red blood cells to non-NK cells, facilitating the separation of NK cells through density gradient centrifugation. (**B**) Median with the interquartile range of the number of NK cells that were purified under the application of the RosetteSep™ human NK cell enrichment kit using untreated buffy coats (*n* = 14). (**C**) Percentage of NK cells after isolation and after 20 days of cultivation (*n* = 2; mean ± SEM). (**D**) NK cell expansion after 20 days of cultivation (*n* = 2; mean ± SEM). (**E**) Number of NK cells after 20 days of cultivation (*n* = 2; mean ± SEM).

Since we had selected the best medium for the cultivation of NK cells, we then analyzed an alternative selection method, namely direct purification of NK cells from buffy coats using the RosetteSep™ human NK cell enrichment kit (Figure 2A). In an attempt to reduce reagent costs as far as possible, we applied this kit to buffy coats as obtained from the blood bank (untreated buffy coats) and two-fold concentrated buffy coats (as explained in Section 2.3). Using this kit, a median of 0.6 × 10^6^ NK cells per milliliter of untreated buffy coat (Figure 2B) was purified. The flow cytometric analysis revealed an average NK cell purity of around 80% for cells isolated out of untreated buffy coats (R) (Figure 2C and Figure S8). However, when the buffy coat was (two-fold) concentrated beforehand (RC), the NK cell purity was reduced to an average of 48.4%. For all conditions (R, RC, and PBMCs), a considerable increase in NK cells was determined after 20 days of cultivation. On day 20, for both conditions where the RosetteSep™ human NK cell enrichment kit was applied, an average NK cell purity of more than 94% was achieved. After 20 days of cultivation, PBMCs also showed increased NK cell proportions, around 44% on average. Regarding the NK cell expansion, this condition (PBMCs) performed best with an around 90-fold expansion of NK cells (Figure 2D). For the other two conditions, the NK cell expansion was much lower. After 20 days of cultivation, the cells purified out of an untreated buffy coat (R) had on average a 17.5-fold NK cell expansion. With a 28.2-fold NK cell expansion on average, a higher NK cell proliferation was observed for cells isolated from a two-fold concentrated buffy coat (RC). However, with an average of 32.4 × 10^6^ cells, this condition (RC) revealed the highest number of NK cells followed by the cells purified out of an untreated buffy coat (R) with an average of 29.8 × 10^6^ NK cells after 20 days of cultivation (Figure 2E). Although the highest NK cell expansion was determined for the cultured PBMCs, this condition showed the lowest number of NK cells with an average of 11.3 × 10^6^ cells.

### 3.3. Human AB Serum Induced the Highest Proliferation in Primary Human NK Cells

**Figure 3:**
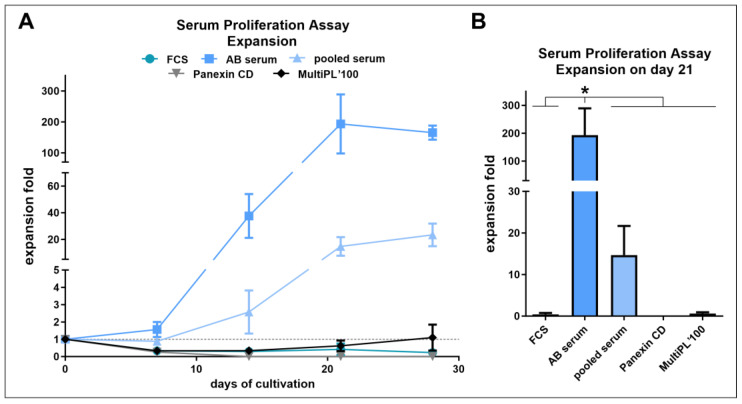
Freshly isolated NK cells were cultured in NK MACS^®^ medium supplemented with IL-2 (500 U/mL) and either FCS, human AB serum, human pooled serum from different blood types, MultiPL’100 or Panexin CD (see figure legend; *n* = 3; mean ± SEM). In one of the three assays performed, NK cells isolated from two donors were mixed. Only living cells were counted to determine the expansion. (**A**) NK cell expansion during the cultivation period using different sera or serum replacements. (**B**) NK cell expansion after three weeks of cultivation using different sera or serum replacements. The statistical analysis was performed per one-way ANOVA, comparing AB serum with every other group, applying Dunnett’s correction for multiple comparisons (ns = not significant; * *p* < 0.0332).

NK cells were isolated from healthy donors and cultured for four weeks to investigate the effect of different sera or serum replacements on cell proliferation (Figure 3). The determination of the expansion revealed that cells supplemented with human AB serum achieved the highest expansion in week three with a 193-fold ± 135 expansion. Here, the cells cultured with human pooled serum showed the second-highest proliferation peak, but only with 15-fold ± 10 expansion. None of the other sera or replacements tested showed an expansion factor greater than 1. Expansion folds of less than 1 indicated that no proliferation was observed for cells cultured in FCS, MultiPL’100, and Panexin CD. With almost no cells left for further cultivation after only two weeks, cultivation with Panexin CD performed the worst. At its optimum at week three, expansion in AB serum-supplemented medium performed significantly better than the other sera or serum replacements, AB serum was chosen for the subsequent experiments.

### 3.4. Positive Effects of NK Cell Activation/Expansion Beads Were Only Observed in Longer Cultivation Periods

**Figure 4:**
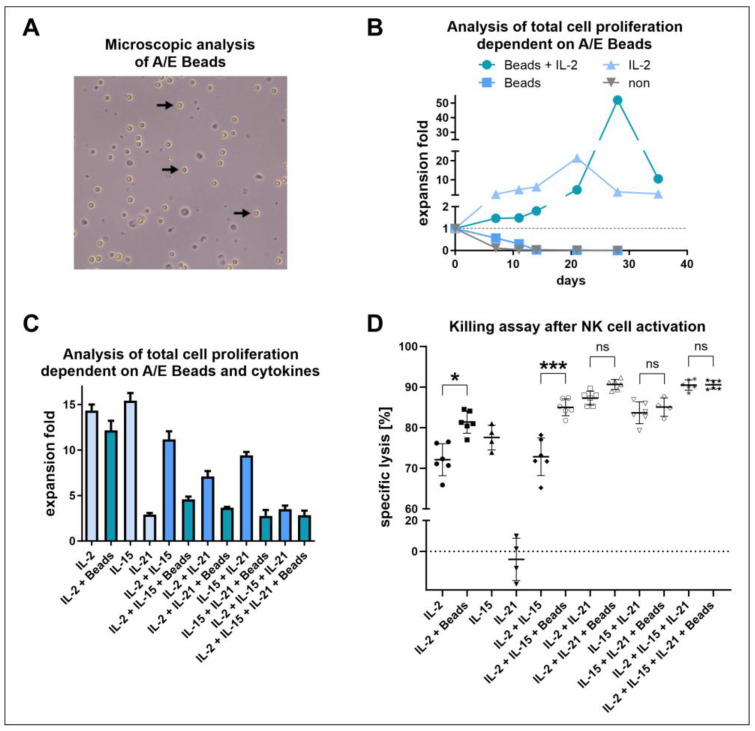
Freshly isolated NK cells were cultured in NK MACS^®^ medium supplemented with human AB serum with different stimulatory factors and their proliferation, as well as cytotoxic potential were determined. (**A**) Arrows pointing at activation/expansion beads (A/E Beads), microscopic image (400×). (**B**) Expansion of NK cells cultured with A/E Beads and IL-2 (500 U/mL), with either A/E Beads or IL-2 (500 U/mL) only or without stimulatory factors. (**C**) Expansion of NK cells stimulated with the cytokines IL-2 (500 U/mL), IL-15 (100 U/mL), or IL-21 (1 U/mL) as well as with the A/E Beads for 10 days in different combinations. (**D**) Specific lysis of target cells determined in a killing assay performed with the NK cells shown in Figure 4C as effector cells and K562 cells as target cells (effector:target = 1:1). The symbols represent technical replicates and the bars represent the mean ± SD (ns = not significant; * *p* < 0.0332; *** *p* < 0.002). The statistical analysis was performed per one-way ANOVA, comparing each group with every other group, and applying Tukey correction for multiple comparisons; however, only selected comparisons are indicated.

To simulate the presence of transformed cells, the influence of activation/expansion beads, stimulating CD2 and NKp46, on NK cell proliferation was determined (Figure 4A,B). Therefore, NK cells were isolated and cultivated in NK MACS^®^ medium supplemented with human AB serum in the presence or absence of IL-2 and activation/expansion beads for 35 days. When NK cells were cultivated without the addition of the cytokine IL-2, almost no cells were left for further cultivation after only two weeks. After three weeks of cultivation, the highest expansion was observed when the cells were supplemented with only IL-2 (21.5-fold expansion). However, from that time point onwards a stronger proliferation was determined when both IL-2 and the activation/expansion beads were applied (52-fold).

### 3.5. IL-2 and IL-15 Alone Induced Higher Proliferation Compared to IL-21 or Combinations, including the Application of NK Cell Activation/Expansion Beads

Since stimulatory factors are essential for NK cell activation, various cytokines and activation/expansion beads were applied in different combinations and the expansion, as well as the cytotoxicity against K562 cells, were determined (Figure 4C,D). After ten days of cultivation, the highest expansion folds were observed for NK cells stimulated with IL-2 or IL-15, although the expansion fold of IL-15 (15.4-fold) was slightly higher than that of IL-2 (14.3-fold) stimulated cells. Among the conditions in which only cytokines were applied, the application of IL-21 alone (2.9-fold) and the combination of IL-2, IL-15, and IL-21 (3.5-fold) induced the weakest proliferation. The expansion of the other cytokine combinations was intermediate between the conditions mentioned before (7- to 11-fold). The addition of activation/expansion beads led to reduced proliferation in all conditions. An effect was especially pronounced when IL-15 was applied in combination with IL-2 (from an 11.2-fold to 4.6-fold expansion) or IL-21 (from a 9.4-fold to 2.8-fold expansion). Regarding the cytotoxicity of NK cells, an opposite effect was observed in the killing assay. Here, stimulation with beads led to a significant increase in specific lysis when treated with IL-2 (from 72.1 ± 3.6% to 81.4 ± 2.5%), as well as IL-2 and IL-15 (from 72.8 ± 4.2% to 85 ± 1.9%). When only IL-21 was applied, the determined specific lysis was nearly absent. However, when IL-21 was combined with other cytokines, no significant increase in specific lysis was achieved by the addition of activation/expansion beads. Consequently, the highest specific lysis was observed under conditions where either IL-21 or beads were used in combination with other cytokines.

### 3.6. Analysis of Activating NK Cell Receptors Revealed Elevated Levels of NKp46, NKG2D, and ICAM-1, Coupled with Reduced Expression of CD16

**Figure 5:**
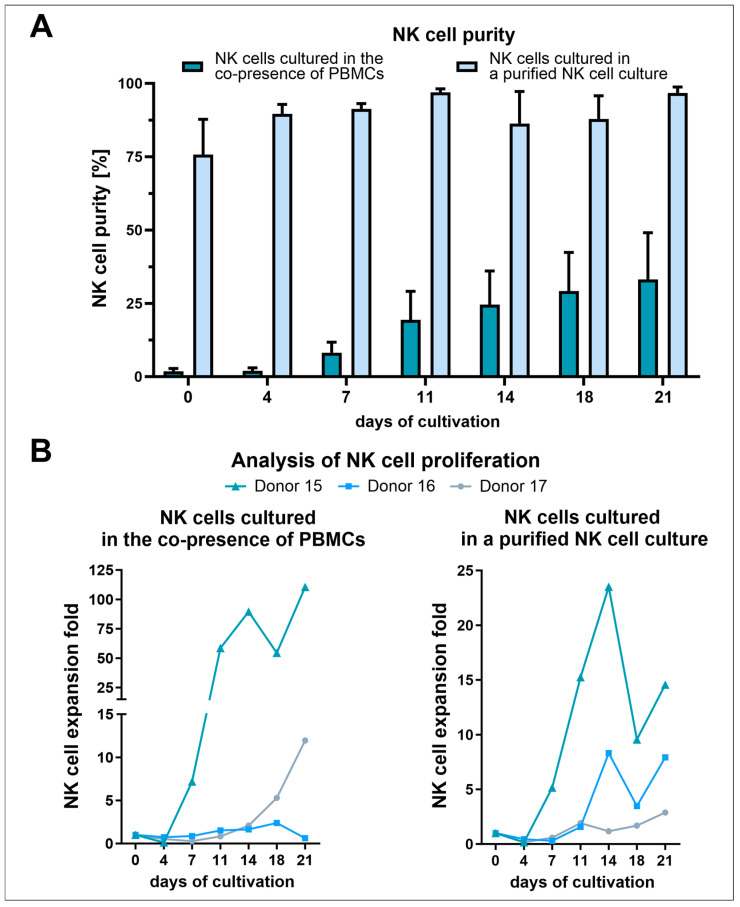
Freshly isolated PBMCs and NK cells were cultured in NK MACS^®^ medium supplemented with human AB serum and 500 U/mL IL-2 for 21 days. (**A**) Percentage of NK cells cultured in the presence of other PBMCs and in a RosetteSep™ human NK cell enrichment kit purified culture (*n* = 3; mean ± SEM). (**B**) NK cell expansion fold in the PBMC culture (left) and the RosetteSep™ human NK cell enrichment kit purified NK cell culture (right) (*n* = 3; mean ± SEM).

To examine the variation in the expression levels of activating receptors on NK cells, we established a flow cytometry panel including markers such as CD45, CD3, CD56, CD16, NKp46, NKG2D, and ICAM-1. NK cells were isolated from buffy coats of three distinct donors using the RosetteSep™ human NK cell enrichment kit. The isolated NK cells and PBMCs were then cultured in NK MACS^®^ medium, supplemented with human AB serum and IL-2, over a three-week period. Following isolation, the initial purity of NK cells in the PBMC culture was approximately 2% during the first four days (Figure 5A). In contrast, cells treated with RosetteSep™ demonstrated a starting purity of 75%. From the fourth day forward, the purity of the NK cell cultures exhibited an increase, achieving a peak purity of 97% starting on day 11. Meanwhile, the co-culture with PBMCs displayed a consistent and more rapid increase in NK cell purity, reaching a peak of 33% after 21 days. When comparing the expansion rates of both cultures, the co-culture with PBMCs resulted in a considerably higher NK cell expansion compared to the cultures of purified NK cells alone (Figure 5B). Specifically, NK cells cultured alongside PBMCs experienced an expansion rate 2 to 3 times higher than that of the purified NK cell cultures. Despite this, a similar growth pattern was observed in both setups: expansion rates decreased after initial seeding, began to increase from day four, experienced another decline on day 18, and then increased once more to the final day of cultivation. The expansion rate of the purified NK cell cultures reached its maximum with an 11-fold increase on day 14, while the NK cells co-cultured with PBMCs reached a peak expansion exceeding a 40-fold increase toward the end of the cultivation period.

**Figure 6:**
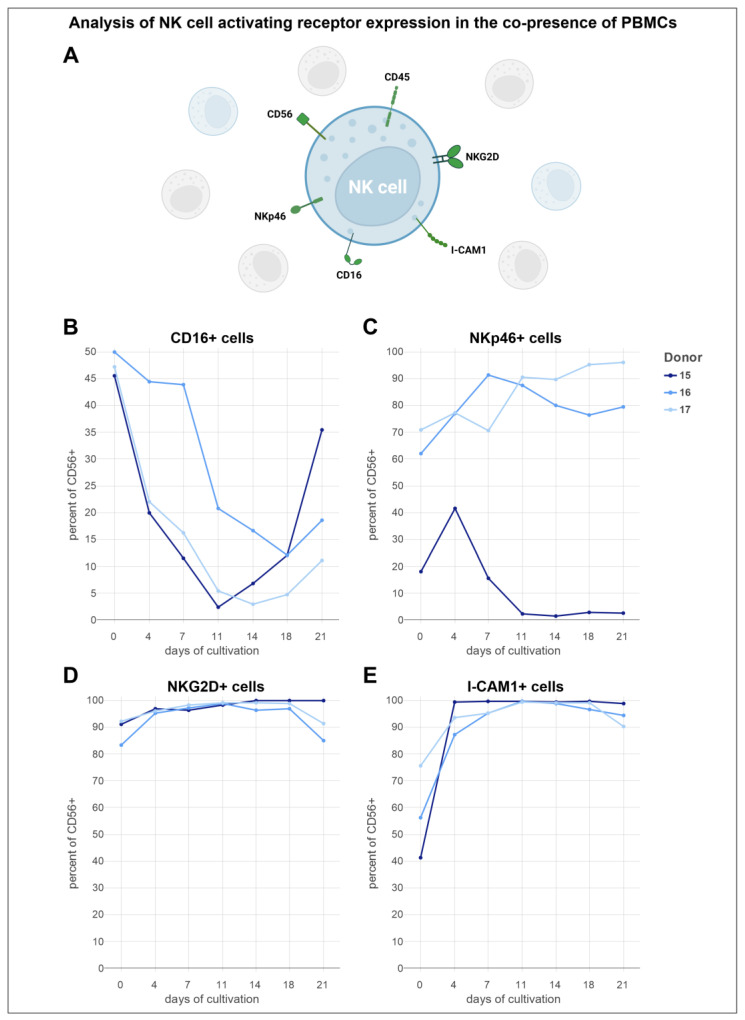
Freshly isolated PBMCs were cultured in NK MACS^®^ medium supplemented with human AB serum and 500 U/mL IL-2 for 21 days (*n* = 3). During the cultivation periods, the receptor expression levels of specific NK cell activation markers were determined through flow cytometric analysis. (**A**) Schematic representation of the expression levels of NK cell activating receptors in the co-presence of other PBMCs. (**B**) Expression levels of the NK cell activation marker CD16. (**C**) Expression levels of the NK cell activation marker NKp46. (**D**) Expression levels of the NK cell activation marker NKG2D. (**E**) Expression levels of the NK cell activation marker I-CAM1.

The expression levels of the receptors CD16, NKp46, NKG2D, and ICAM-1 on NK cells (CD45^+^/CD3^−^/CD56^+^) in cultures of complete PBMCs were assessed (Figure 6A). Although there was substantial donor variability in the expression levels of these activating receptors, consistent expression trends were identified. The initial proportions of CD16^+^ cells among the three donors ranged from 45% to 50% (Figure 6B). For donor 15, the percentage of CD16^+^ cells decreased notably, reaching a low of 2% by day 11 of cultivation, followed by a steady increase thereafter. Donor 16 had the highest initial fraction of CD16^+^ cells at exactly 50%, which began to decrease within the first week. A notable reduction occurred on day 7, reaching a low of around 12% on day 18, before slightly increasing again. Similarly, the trajectory of donor 17 included a decrease during the first two weeks, with the fraction of CD16^+^ cells falling to almost 3% by day 14. Toward the end of the cultivation period, CD16 expression began to rise again. In contrast to the other donors, donor 15 had a notably lower initial proportion of NKp46^+^ cells, with only 18% (Figure 6C). This proportion rose sharply, peaking at over 41% on day 4, followed by a gradual decline to a low of 1.5% by day 14. A minor recovery was noted from days 14 to 18, before a slight decrease toward the end of the cultivation period. Conversely, NKp46^+^ cells obtained from donor 16 surged from 62% to 91% within the first week, then gradually declined over the following 11 days before showing a modest recovery. Donor 17 started with the highest baseline of over 70% NKp46^+^ cells, which increased in the first 4 days, and then decreased until day 7. Afterward, the trajectory mirrored that of donor 16 with a sharp increase, followed by a drop on day 14, and then a rise toward the end of the cultivation period, ultimately achieving a peak of over 96%. The NKG2D expression from cells obtained from donor 15 began at an initial 91%, which approached a peak of nearly 100% by the end of the cultivation period (Figure 6D). For donor 16, the initial proportion of NKG2D^+^ cells was documented at 83%. An increase in this fraction was recorded until day 4, followed by a marginal rise, and then a reduction on day 14. An upward tendency in the NKG2D^+^ cell proportion was noted thereafter, which then diminished toward the end of cultivation. Donor 17 had the highest initial proportion of NKG2D^+^ cells at 92%, showing an increase over the first two weeks of cultivation. This growth was initially more pronounced and then leveled off in the second week. The proportion of NKG2D^+^ cells for donor 17 eventually reached 99%, before decreasing to 91% toward the end of the cultivation period. The ICAM-1^+^ cell fractions trajectory exhibited a similar trend across different conditions (Figure 6E). Notably, donor 15 began with the lowest initial ICAM-1^+^ cell proportion of around 40% but experienced the most rapid increase, nearing 100% by day 4—a level that was sustained for the remainder of the cultivation period. Donors B and C initiated with cell fractions ranging from 56% to 75%, both exhibited an increase in the proportion of ICAM-1^+^ cells from the start of the cultivation, reaching nearly 100% by day 11. The fraction of ICAM-1-expressing cells derived from donor 16 underwent a gradual decrease during the final 10 days, whereas donor 17 maintained a steady level of ICAM-1^+^ cells, akin to donor 15, before a noticeable decrease in cell fraction toward the end of cultivation.

**Figure 7:**
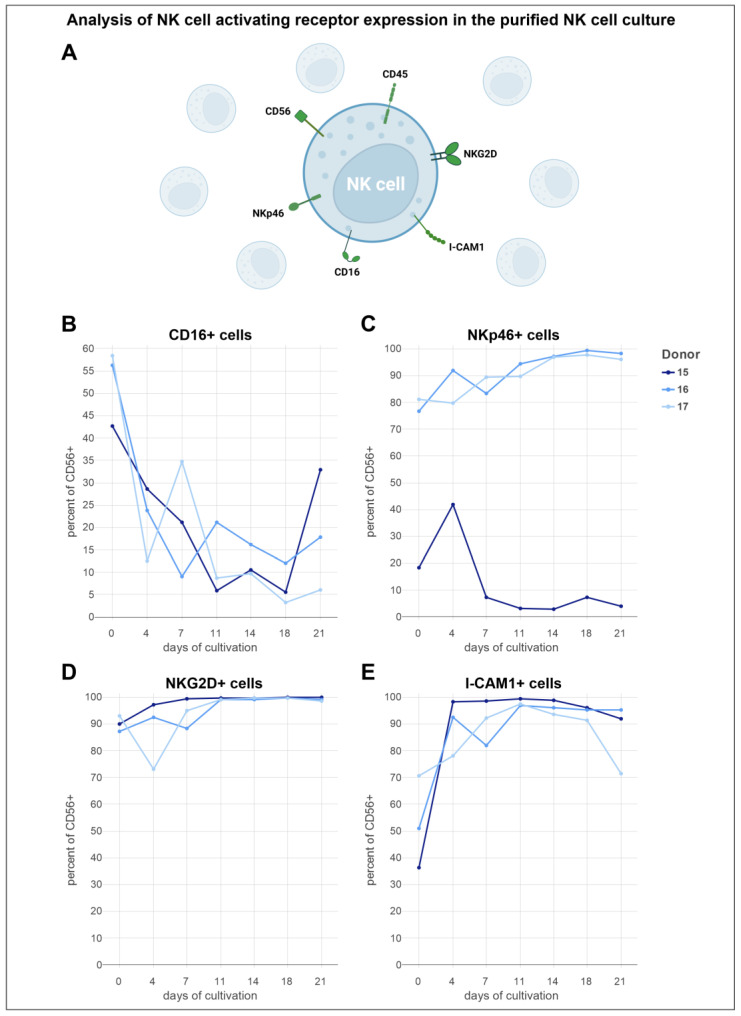
Freshly isolated and purified NK cells were cultured in NK MACS^®^ medium supplemented with human AB serum and 500 U/mL IL-2 for 21 days (*n* = 3). During the cultivation periods, the receptor expression levels of specific NK cell activation markers were determined through flow cytometric analysis. (**A**) Schematic representation of the expression levels of NK cell activating receptors in the absence of other PBMCs. (**B**) Expression levels of the NK cell activation marker CD16. (**C**) Expression levels of the NK cell activation marker NKp46. (**D**) Expression levels of the NK cell activation marker NKG2D. (**E**) Expression levels of the NK cell activation marker I-CAM1.

To compare the expression levels of the aforementioned receptors, they were also assessed in purified NK cell cultures using the same flow cytometric panel and donors (Figure 7A). The patterns observed in the trajectories of activation marker expressions displayed minimal variation in comparison to NK cells cultured in the presence of other PBMCs. For donor 15, compared to the other donors, the initial proportion of CD16-expressing cells was observed to be the lowest at 43% (Figure 7B). This fraction experienced a decrease over the first 11 days, followed by fluctuations in the next 7 days, ultimately resulting in a reduced fraction of nearly 6%. A subsequent increase in this proportion was noted after 18 days. For donor 16, the trajectory for the CD16^+^ cell proportion mirrored that of donor 15, starting at 56%. However, a notable difference was that the minimum fraction of donor 16 dropped to 9% by day 7. Donor 17 started with the highest proportion of CD16^+^ cells at 58%, experiencing a marked drop within the first 4 days. This was followed by a peak on day 7 and a consistent decline toward the end of cultivation, culminating in a low of 3% on day 18. A notable trend in the NKp46^+^ cell fraction was observed for two of the donors (Figure 7C). Donor 15 had the lowest initial NKp46^+^ cell proportion at 20%, which began to increase immediately after cultivation, reaching a peak at 42% on day 4. Following this peak, a decline led to a minimum cell proportion of just under 3%. Toward the latter stages, minor fluctuations were noted. In contrast, the NKp46^+^ cell proportion of donor 16 began at approximately 77%, showing a mostly steady increase, despite occasional dips after the first and third weeks of cultivation. A peak of over 99% NKp46^+^ cells was reached by day 18. Donor 17 started with an NKp46^+^ cell fraction similar to the initial value of donor 16. However, a decrease to a minimum of 78% was observed for donor 17, followed by a steady increase, reaching a population of 98% NKp46^+^ cells between days 14 and 18, before declining as cultivation neared the end. The proportion of NKG2D^+^ cells from donor 15 showed a steady increase during the first 18 days of cultivation, starting from an initial 90% and reaching close to 100% by day 11, where it remained until the end of cultivation (Figure 7D). In contrast, the NKG2D^+^ cell fraction from donor 16, with an initial 87%, experienced early fluctuations. From day 7, a consistent rise was noted, leading to a stable plateau for the latter half of the cultivation period, with a maximum cell fraction exceeding 99% on day 18. Notably, the NKG2D^+^ cell proportion of donor 17 began at 93% but experienced a sharp drop to 20% within the first four days, marking a clear departure from the trends observed in donors A and B. However, this decrease was followed by a sharp rise to 95% by day 7, which then leveled off until day 18, before a slight decline. On day 18, the cell fraction of donor 17 also reached nearly 100%. The ICAM-1^+^ cell fraction of RosetteSep™ purified NK cells obtained from donor 15 showed a remarkable increase, jumping from an initial 36% to 98% in just four days (Figure 7E). This level experienced a minor rise over the following seven days, reaching a peak above 99%. A gradual decline in the cell proportion of donor 15 was noted from day eleven to day 21. Donor 16 initially mirrored the growth pattern of donor 15 with a strong increase in the ICAM-1^+^ cell fraction from 51% to over 90% in the first 4 days. A slight decrease was observed on day seven, but the cell fraction peaked at 97% by day eleven, followed by a slow decrease thereafter. For donor 17, the ICAM-1^+^ cell proportion showed a more gradual increase until day 11, achieving the same peak as donor 16 on that day, before declining toward the end of the cultivation period. In addition to the observed similarities of the positive cell fractions, the mean fluorescence intensity of both the purified NK cells and NK cells in the PBMC cultures ranged on the same scale (Figures S9 and S10).

### 3.7. NK Cells Cultivated in NK MACS^®^ Medium Supplemented with Human AB Serum and Stimulated with IL-2 Potently Target and Lyse Primary Glioblastoma Cancer Stem Cells

**Figure 8:**
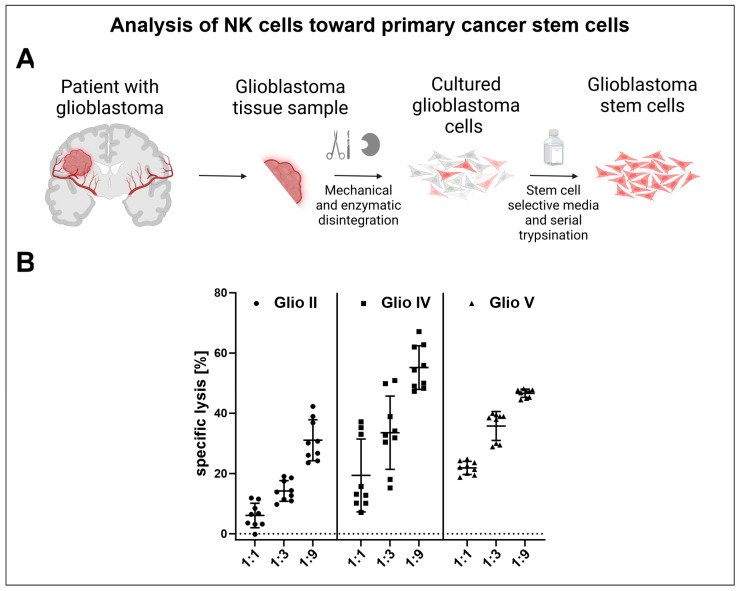
NK cells cultivated in NK MACS^®^ medium supplemented with human AB serum and IL-2 potently kill primary glioblastoma cancer stem cells from different donors. Adapted from [55]. (**A**) Glioblastoma tissue samples were minced and incubated with proteolytic enzymes and cultured under application by serial trypsinization to generate a cancer stem cell fraction. (**B**) Specific lysis of target cells determined in a killing assay performed with the activated NK cells as effector cells and glioblastoma cancer stem cells as target cells (effector:target = 1:1, 1:3, 1:9). The symbols represent technical replicates and the bars represent the mean ± SD.

To determine whether primary NK cells, cultured in NK MACS^®^ medium supplemented with human AB serum and IL-2, have the cytotoxic capacity to effectively lyse primary cancer stem cells, killing assays were conducted using different glioblastoma cancer stem cells as target cells. Primary cancer stem cells were characterized in our earlier publication we used different amounts of NK cells in comparison to cancer stem cells [55]. As expected, efficient killing was observed, with mean specific lysis values ranging from 6.1% to 21.9% for the 1:1, 14.2% to 35.8% for the 1:3, and 31.1% to 55.2% for the 1:9 target-to-effector ratio (Figure 8). Taken together, these data exemplify that cultivation of NK cells in the determined conditions generates highly activated NK cells able to efficiently kill cancer stem cells.

## 4. Discussion

This study aimed to develop a robust and cost-effective method for the enrichment and expansion of human NK cells to produce high yields of activated NK cells for in vitro assays and as a foundation for developing cancer immunotherapy protocols. Initially, we investigated an adherence-based selection method for NK cell enrichment using different culture media. Although this approach did not achieve our goals, one of the tested media proved vastly superior, resulting in mostly high-purity NK cell cultures even with this suboptimal protocol. Recognizing the importance of culture medium selection, we further characterized NK cell culture in this medium. We tested the RosetteSep™ human NK cell enrichment kit as an alternative enrichment method and assessed the effects of various sera and serum replacements, cytokines, and stimulatory beads with this medium. After establishing suitable culture conditions, we examined the expression levels of activating NK cell receptors throughout the culture period and studied how the presence of other autologous PBMCs influenced these receptors. Cells cultured in these conditions demonstrated potent cytotoxicity against primary human glioblastoma cancer stem cells.

Unexpectedly, in the selection step of the adherent selection protocol, the serum-free medium apparently led to stronger adherence of both total and NK cells. Serum contains a multitude of adherence factors, whereas chemically defined serum-free media typically only include a limited number [56,57]. In contrast, the use of DMEM/F12 and NK MACS^®^ led to a loss of the majority of seeded NK cells. Still, the culture of these remaining NK cells for 20 days in NK MACS^®^ led to robust NK cell expansion and purification. The use of DMEM/F12 and PRIME-XV NK Cell CDM resulted in low NK cell numbers and low contents. Conclusively, the markedly superior performance of NK MACS^®^ medium supplemented with 5% human AB serum presents it as a robust choice for NK cell cultivation, especially in suboptimal conditions. As we excluded purely donor-dependent effects using cryopreserved cells and there is limited value in characterizing unsuitable medium, a small-scale direct medium comparison with the same donors was not performed. In essence, the optimized commercially available NK cell media supported cell growth much better than the standard medium DMEM/F12. The less complex nature of chemically defined medium probably leads to a smaller optimal operating window, hampering the performance of PRIME-XV NK Cell CDM. DMEM/F12, in essence, might be a standard medium considered “rich”, but standard media are mostly based on traditional modifications of minimal media, rather than optimized [58,59]. Although we did not directly characterize the composition of the adherent cell fraction, the presence of some CD3 positive and CD3 and CD56 double negative cells on day 20 speaks for its heterogeneity. Besides NK cells, monocytes are also reported to become adherent, but in contrast to T cells, have nearly no positive feeder cell effect [60,61]. Overall, the adherent selection protocol seems to rely on a weak enrichment of NK cells and possibly a moderate reduction of the T cell content through the removal of non-adherent cells, as well as the outgrowth of NK cells due to high doses of IL-2 with possible anti-T cell cytotoxicity of the activated NK cells [62]. In the context of clinical application, contamination with T cells implies an increased risk of GVHD or cytokine release syndrome [63,64]. In conclusion, the use of adherent selection with the only medium inducing robust NK cell expansion leads to a loss of NK cells but still fails to effectively deplete T cells. Therefore, we investigated the RosetteSep™ human NK cell enrichment kit as an alternative to adherent NK cell enrichment.

Purification of NK cells using the tetrameric antibodies in the RosetteSep™ human NK cell enrichment cocktail is convenient and timesaving compared to other isolation methods like magnetic cell sorting. This method involves adding the cocktail before density gradient centrifugation, making NK cell enrichment require minimal extra effort beyond PBMC isolation. This approach resulted in high NK cell purities with minimal T and NKT cell contamination. Given the higher cost of the RosetteSep™ cocktail compared to the adherent selection, buffy coats were concentrated to reduce expenses. Concentrating the buffy coat two-fold adhered to the manufacturer’s recommendations regarding nucleated cell-to-red blood cell ratio and cell density. The reduced NK cell purity post isolation from concentrated buffy coats in comparison to untreated buffy coats was compensated by faster proliferation in the 20-day cultivation period. The effect was even more pronounced when PBMCs were directly cultivated, demonstrating that the presence of other PBMCs positively influences NK cell proliferation. Autologous and allogeneic PBMCs are often used as feeder cells, but they are usually growth-inhibited to avoid interfering with NK cell proliferation [41,65]. Here, high NK cell expansion rates were achieved by cultivation in NK MACS^®^ medium with 5% human AB serum without inhibiting PBMC growth. While larger expansions of NK cells were achieved in the presence of other PBMCs, higher final NK cell numbers were obtained when starting with purified NK cells before the 20-day cultivation period, when normalized to total seeding cell count. This finding must be considered when selecting culture vessel size and calculating media consumption in large-scale approaches.

While 5% human AB serum is the manufacturer’s recommendation of supplementation for NK MACS^®^ medium, other sera or serum replacements have been applied to NK cell culture. To test whether changing this component can reduce costs, enhance performance, or address regulatory and ethical concerns, we conducted a comparison. Specifically, we compared human AB serum, pooled human serum from various blood types, FCS, the commercially available human platelet lysate MultiPL’100, and the chemically defined serum replacement product Panexin CD. Regarding proliferation, human AB serum vastly outperformed all tested alternatives. Notably, only the human serum variants induced proliferation in the early stages of culture. Although FCS-based NK cell culture is functional in several protocols using twice the amount (10%) [66,67], FCS is of concern for clinical use due to its animal origin and ethical considerations. Combined with its low performance, further investigation for NK cell expansion is obsolete [68]. Meggyes et al. also demonstrated increased NKG2D and perforin expression in cells cultured in human AB serum compared to FCS, suggesting enhanced cytotoxic activity [69]. The superiority of AB serum over pooled serum may be attributed to the absence of donor-specific antibodies [70]. As MultiPL’100 demonstrated an increase in cell number in the later stage of culture only, a protective impact of human platelet lysate on the viability of NK cells over extended culture periods might be investigated for long-term cultures. Panexin CD, a chemically defined serum replacement product, not only failed to support NK cell expansion but also to maintain viability for more than two weeks when used directly after isolation. Gradual transfer to the chemically defined environment might yield different results, especially with autologous serum from the cell’s donor [71]. This approach could also apply to the chemically defined PRIME-XV NK Cell CDM medium. However, incorporating serum to facilitate adaptation to a chemically defined, serum-free medium presents an inherent contradiction; it undermines the regulatory benefits associated with using chemically defined supplements, which are designed to ensure consistency and animal-free compliance in cell culture conditions.

In the same assay, we further evaluated whether NK cells from different donors can be combined to achieve the large number of cells required for therapeutic use. Therefore, we mixed isolated NK cells from two donors in one of three assays performed to identify the most beneficial serum or serum replacement. As the results indicated no major differences compared to the other assays, we conclude that combining NK cells from different donors in the same culture may be feasible.

With a suitable base medium and serum identified, we next investigated the effect of activation/expansion beads carrying agonistic anti-NKp46 and anti-CD2 antibodies on NK cell proliferation. These should simulate the presence of transformed cells. As discussed above in the context of ionomycin and IL-2 use in the protocol for adherence-based NK cell isolation, optimal NK cell expansion needs both a primary mitogenic stimulus and costimulatory signals. The primary mitogenic stimulus is provided by a cytokine, e.g., IL-2, and the costimulatory signal lastly seems to be mediated through calcium flux. As the combination of NKp46 and CD2 agonism was shown to induce calcium flux, it can constitute the costimulatory signal enhancing proliferation. Therefore, as expected, the application of the activation/expansion beads in conjunction with IL-2 had a positive effect during longer cultivation periods, compared to IL-2 alone. However, during shorter culture durations proliferation was inhibited. The enhancement of proliferation only became apparent after four weeks, while most protocols for NK cell expansion do not exceed three weeks [26,72,73]. The proliferation reduction in the early phase is probably induced through NK cell autolysis, as previously reported for T11/CD2 or CD16 activation. Furthermore, the combination of the IL-2 signal with the calcium flux over time leads to the upregulation of both IL-2Rα and IL-2Rβ chains [32,36]. Thus, the responsiveness to IL-2 could increase over time, explaining the enhanced proliferation in the later stage. In addition, Casmir de Rham and colleagues previously demonstrated the ability of IL-2 to induce NKp46 expression, potentially creating a positive feedback loop [46].

With the overall importance of the mitogenic stimulus through cytokines alone, and the interplay with NK cell activating receptor agonism, we explored various combinations of cytokines and activation/expansion beads. In the literature, IL-2 and IL-15 are often considered to be equally effective due to their partly shared pathways. As a result, IL-2 is usually preferred for its cost efficiency [74]. Our results show that stimulation with IL-15 exhibited slightly superior performance compared to IL-2 in terms of expansion and cytotoxic abilities. This aligns with the findings of Mao et al., who also demonstrated the superiority of IL-15 over IL-2 in terms of cytotoxicity [75]. Neither the combination of IL-2 and IL-15 nor any of the other combinations of cytokines yielded better or comparable proliferation to IL-2 or IL-15 alone. In particular, the addition of activation/expansion beads and/or IL-21 consistently led to a reduction in expansion, as earlier noticed for the combination with IL-2. One of the main findings observed in the cytotoxicity assay was that activation/expansion beads can significantly augment NK cell cytotoxicity. However, a distinct effect of the beads on the cytotoxic abilities was only evident in the absence of IL-21. Hence, to achieve optimal cytolytic activity, the application of IL-21 with either IL-2 or IL-15 is expected to yield the best outcome, as the addition of beads had a more pronounced negative effect on cell proliferation. As Rham et al. demonstrated, the cytokines IL-2, IL-15, and IL-21 amplify the expression levels of various receptors differently [46]. Therefore, it can be assumed that NK cells cultured with distinct cytokines may exhibit greater variation in activating receptors [46,76]. As the cheaper cytokine with similar expansion to IL-15 and the negative effects of IL-21 on proliferation, we determined IL-2 alone, with its slightly lower cytotoxicity, as well suitable.

Both IL-21 and activation/expansion beads lead to reduced proliferation but enhanced cytotoxicity, indicating a tradeoff between proliferation and optimal cytotoxicity. A comparison study of NK cell expansion with genetically modified feeder cells expressing membrane-bound (mb) IL-15 or mbIL-21, alongside other stimulating ligands, highlighted a correlation between NK cell function or proliferation and their metabolic profile. mbIL-15 led to superior functionality, while mbIL-21 primarily enhanced proliferation. Essentially, both profiles were inducible in sequence but not in combination. Functionally superior mbIL-15 stimulated cells exhibited higher baseline levels and spare respiratory capacities of oxygen consumption rate and extracellular acidification rates, compared to mbIL-21 stimulated cells. Furthermore, glycolytic capacity, ability to replenish ATP from glycolysis, and glucose uptake were enhanced in mbIL-15 stimulated cells. However, mbIL-15 expanded cells were less dependent on glycolysis for cytotoxic function compared to mbIL-21 expanded cells. Overall, mbIL-15 stimulated cells were found to be more energetic, resembling stressed NK cells, while mbIL-21 stimulated cells resembled quiescent, resting NK cells, in comparison to various populations stimulated with feeder cells [77]. These data show a clear distinction between a cytotoxic metabolic profile and a proliferative metabolic profile, suggesting a tradeoff between optimal expansion and cytotoxicity, as both metabolic profiles could not be combined. However, stimulation with IL-15, compared to resting NK cells, leads to both proliferation and enhanced cytotoxicity, inducing mTOR and increasing glycolysis and oxidative phosphorylation [78]. Additionally, our data show that IL-15, compared to IL-2, further enhances both effects in combination, indicating that both effects can coexist to a certain extent. The effects of soluble cytokines and membrane-bound or otherwise immobilized cytokines may differ, and the influences of the applied feeder cells must be considered. In this context, short-term stimulation with IL-21 has been shown to enhance both proliferation and cytotoxicity, while prolonged exposure induces cell death [79,80,81]. Feeder cells are often susceptible to lysis and thus may disappear within a few days, suggesting that transient soluble IL-21 stimulation might be a better comparison, possibly explaining the observed differences. However, immobilized IL-21 on magnetic beads could not be substituted by soluble IL-21, indicating further differences [82]. Despite the induction of cell death, prolonged IL-21 stimulation led to enhanced cytotoxicity due to granzyme B upregulation [81]. As discussed above, activation/expansion beads might have reduced the early cell count and thus expansion through NK cell autolysis. The common gamma chain cytokines IL-2, IL-15, and IL-21 primarily signal through JAK-STAT pathways, PI3K-Akt, and RAS-MAPK [47,48]. While activating NK cell receptor signaling has largely different upstream components, downstream, PI3K-Akt and RAS-MAPK are also activated [83]. Additionally, the DAP10 cytoplasmic domain, for example, contains a YINM motif which, far downstream, also leads to STAT5 phosphorylation, among others [84]. Therefore, by interacting signaling pathways, activating NK cell receptors can lead to similar transcription protocols as cytokines. For instance, NKp46 signaling and CD2 signaling include RAS-MAPK, PI3K-Akt, and NF-kB [85,86]. Thus, while there is evidence of a metabolic tradeoff between proliferation and cytotoxicity, in the applied setting, cytotoxic effects on NK cells are most likely the reason for lowered expansion.

Importantly, a correlation between NK cell-activating receptor expression and cytotoxicity has been shown [87]. To assess the impact of other PBMCs on the activation status and cytotoxic potential of NK cells under our determined most favorable conditions (NK MACS^®^ medium supplemented with human AB serum and 500 U/mL IL-2), we used an 8-color flow cytometry panel. This panel incorporates DAPI, CD45, CD3, and CD56 to identify living NK cells and the activating receptors CD16, NKp46, NKG2D, as well as ICAM-1. Both isolated NK cells, enriched using the RosetteSep™ human NK cell enrichment kit, and PBMCs were cultivated over a period of three weeks for this investigation. As expected, due to the already mentioned feeder cell effect of PBMCs, purified NK cells demonstrated a considerably lower level of expansion compared to those co-cultured with PBMCs. As observed above, the chosen culture conditions favored NK cell expansion over other cell types, raising the NK cell percentage in the PBMC culture. The purified NK cell culture in this way reached nearly 100% after only 11 days of culture.

Interestingly, in this experiment, donor 15 showed by far the largest expansion and displayed an activating receptor profile markedly different from the other donors. This profile was characterized by a strongly reduced NKp46 expression and a peak of ICAM-1 expression in early culture. Close examination of the flow cytometry data reveals that the CD56^bright^ NK cell content mostly disappeared after day 7, whereas the other donors predominantly shifted to a CD56^bright^ phenotype. As CD56^bright^ NK cells normally express the highest levels of NKp46, the loss of these cells in donor 15 explains the shift to lower NKp46 expression [88]. While CD56^bright^ NK cells are reported to strongly proliferate even with low amounts of IL-2, CD56^dim^ NK cells proliferate much less, even in response to high levels of IL-2 [89]. Furthermore, unlike in donors 16 and 17, and contrary to the literature, the induction of ICAM-1 expression was primarily detected in the CD56^dim^ subset [90]. Since ICAM-1 has been suggested as an activation marker and has stimulatory properties in an autoregulatory positive feedback loop through LFA-1, it is evident that these CD56^dim^ NK cells were potently stimulated. The CD56^bright^ NK cell death might be explained by accumulated DNA damage and replication stress before culture, possibly due to an immune challenge since this NK cell subset was shown to be more prone to replication stress-induced apoptosis [91]. In this scenario, IL-2 stimulation might mediate cytokine-induced apoptosis through autocrine interferon-gamma (IFN-γ) and tumor necrosis factor-alpha (TNF-α) secretion. Both cytokines were also shown to induce ICAM-1 upregulation in turn [92]. 

The Fcγ-receptor CD16 is responsible for ADCC and typically used in the distinction of the main NK cell subsets in the peripheral blood [93]. The observed CD16 downregulation can largely be attributed to the activity of ADAM17. ADAM17 is an enzyme integral to the proteolytic shedding of cell surface proteins. Previous studies have highlighted the role of ADAM17 in the downregulation of CD16 following NK cell activation. ADAM17-mediated shedding of the FcγRIIIA/CD16A results in decreased surface expression of CD16, effectively reducing the ability to mediate ADCC, thereby preventing over-activation of NK cells. The utilization of an anti-ADAM17 antibody or small molecule inhibitor can be considered for inhibition of CD16 shedding in case of combined cancer immunotherapy with antibodies, needing NK cells capable of mediating ADCC. The cell adhesion molecule ICAM-1 has important functions in migration, cellular homing, and development of the immunological synapse. It provides a link to the adaptive immune system by stimulating T cells and has been suggested as an activation marker [17,94]. The observation that PBMCs appear to be slightly beneficial for ICAM-1 expression implies that in—addition to IL-2—other cytokines released from PBMCs, such as IL-1, TNF-α, and INF-γ increase ICAM-1 expression [95]. Overall, the increase just shows potent activation. NKG2D is one of the best-known activating receptors on NK cells. It binds to at least eight different ligands, which are stress-induced or overexpressed in transformed and infected cells [96]. Its observed decline near the end of PBMC cultures may be explained by the secretion of TGF-ß1 or L-kynurenine from other PBMCs as a regulatory mechanism, both having suppressive effects on NKG2D expression [97,98]. The natural cytotoxicity receptor NKp46 is the most evolutionary ancient NK cell activation receptor. NKp46 recognizes some infectious ligands and externalized calreticulin, allowing the elimination of cells with endoplasmic reticulum stress [99]. The high levels of donors 16 and 17 can be attributed to the shift to a CD56^bright^ phenotype, as explained above. Taken together, these results suggest that NK cell cytotoxicity in the applied culture conditions, at least regarding non-ADCC, is the highest between days 7 and 18. ADCC capacity, however, should be most pronounced before day 4 or after day 21. In line with this, we have demonstrated effective lysis of glioblastoma cancer stem cells from different donors through NK cells cultured using this protocol [55].

Overall, a large inter-donor variation in proliferation could be observed. NK cell activity and proliferation are influenced by many factors. Since blood donations are anonymized, we lack information on donor lifestyles. Though from healthy donors, the criteria are broad. For example, obesity and metabolic disorders like diabetes negatively affect NK cell function. Obesity disrupts cellular metabolism, altering NK cell activity and cytokine production [100,101,102]. Positive factors include “forest bathing”, which enhances NK cell function [103,104,105,106], and supplements like ashwagandha and garlic [107]. Age and sex also impact NK cell activity, with younger individuals and specific sex differences showing varying responses [87]. Ethnic background and CMV infection status further influence NK cell function and experimental outcomes [108,109,110]. In summary, multiple intrinsic and extrinsic factors significantly impact NK cell functionality, highlighting the complexity of their regulation.

## 5. Conclusions

In conclusion, NK cell culture in NK MACS^®^ medium supplemented with human AB serum and IL-2 was identified as a robust culture environment, even in suboptimal conditions. NK cell enrichment with the RosetteSep™ human NK cell enrichment kit was fast, easy to perform, and yielded high NK cell purity. The purified NK cells lacked the feeder cell effect associated with the presence of PBMCs, but activating receptor expression levels of CD16, NKp46, NKG2D, and ICAM-1 followed similar patterns and exhibited similar expression levels in both conditions. These NK cells displayed effective lysis of primary glioblastoma cancer stem cells. In summary, we showed that culturing PBMCs or purified NK cells in NK MACS^®^ medium supplemented with human AB serum and IL-2 is suitable for basic laboratory research and might be a good basis for the development of clinical expansion protocols.

## Figures and Tables

**Table 1:** Used fluorochrome-conjugated antibodies and dyes for the identification of the NK cell purity, viability, and T and NKT cell content during the proliferation assays.

Antibody/Dye	Function/Marker
CD3-FITC	T and NKT cell marker
CD56-APC-A700	NK cell marker
DAPI	Viability

**Table 2:** Used fluorochrome-conjugated antibodies and dyes for the NK cell receptor analysis. All antibodies were obtained from Miltenyi Biotec GmbH, Gladbach, Germany.

Antibody/Dye	Function/Marker
CD45-VioGreen	Hematopoietic cell marker
CD3-PE	T and NKT cell marker
CD56-APC-Vio770	NK cell marker
CD16-PE-Vio615	NK cell marker
NKp46-Vio-Bright B515	NK cell marker
NKG2D-PE-Vio770	NK cell marker
ICAM-I-APC	NK cell marker
DAPI	Viability

**Table 3:** Summary of the adherent selection experiment, including the median of the NK cell purity as well as T cell contamination. In addition, the number of NK cells in the isolated PBMCs, as well as enriched and expanded cells on day 20 of cultivation is given. The medium that performed best is highlighted together with the corresponding NK cell purity in bold letters. The complete data are detailed in Table S1.

Medium	NK Cells [%]	T + NKT Cells [%]	Number of NK Cells [×10^6^]
Isolated PBMCs			
PRIME-XV NK Cell CDM (*n* = 4)	7.2%	47.8%	7.2
	(3.4–10.4%)	(46.8–71.8%)	(3.4–10.4)
DMEM/F-12 (*n* = 4)	7.2%	47.8%	7.2
	(3.4–10.4%)	(46.8–71.8%)	(3.4–10.4)
NK MACS^®^ (*n* = 4)	7.3%	62.2%	7.3
	(2.8–12.4%)	(48.7–80.9%)	(2.8–12.4)
Adherently selected and expanded cells on day 20 of cultivation			
PRIME-XV NK Cell CDM (*n* = 4)	7.2%	47.8%	7.2
	(3.4–10.4%)	(46.8–71.8%)	(3.4–10.4)
DMEM/F-12 (*n* = 2)	1.1%	33.5%	<0.1
	(0–2.3%)	(1.1–65.8%)	(0–<0.1)
**NK MACS^®^** (*n* = 4)	**89%** **(39.3–90.2%)**	7.2% (6.5–55.7%)	16.2 (0.4–59)

## Data Availability

Raw data are available upon request from the corresponding author.

## Supplementary Materials

The following supporting information can be downloaded at https://www.mdpi.com/article/10.3390/cells13131148/s1, Figure S1:Representation of the gate settings during flow cytometry analysis to identify NK, NKT, and T cells; Figure S2: Representation of the gate settings during flow cytometry analysis to identify NK cells and characterize them regarding CD16, NKp46, ICAM-1, and NKG2D expression; Figure S3: Microscopic images of freshly isolated, cultured, and adherently selected PBMCs in different media for NK cell enrichment; Figure S4: Flow cytometric analysis of freshly isolated, cultured, and adherently selected PBMCs in different media for NK cell enrichment; Figure S5: Characterization of the adherent selection method regarding removed cell fractions, total cell numbers and mean NK cell expansion after culture; Figure S6: Characterization of the adherent selection method regarding individual donor expansion, removed NK cell fractions, and donor specific NK cell expansion after culture; Figure S7: NK cell purity of adherent selected cells obtained from donor 6 cultured in different media on day 20 of cultivation; Figure S8: NK cell purity after isolation of NK cells using the RosetteSep™ human NK cell enrichment kit and PBMCs; Figure S9: Mean expression levels of activating NK cell receptors on NK cells co-cultured PBMCs; Figure S10: Mean expression levels of activating NK cell receptors on NK cells cultured without PBMCs; Table S1: Summary of the adherent selection experiment, including the median of the NK cell purity as well as T cell contamination.
