# Supplementary material for: A Critical Role of Culture Medium Selection in Maximizing the Purity and Expansion of Natural Killer Cells
Source: Cells. 2024 Jul 5;13(13):1148. doi: 10.3390/cells13131148 (PMC11240826; doi:10.3390/cells13131148)
Supplement: Supplementary file 1 [file cells-13-01148-s001.zip › cells-3058590-supplementary.pdf]

Supplementary Materials

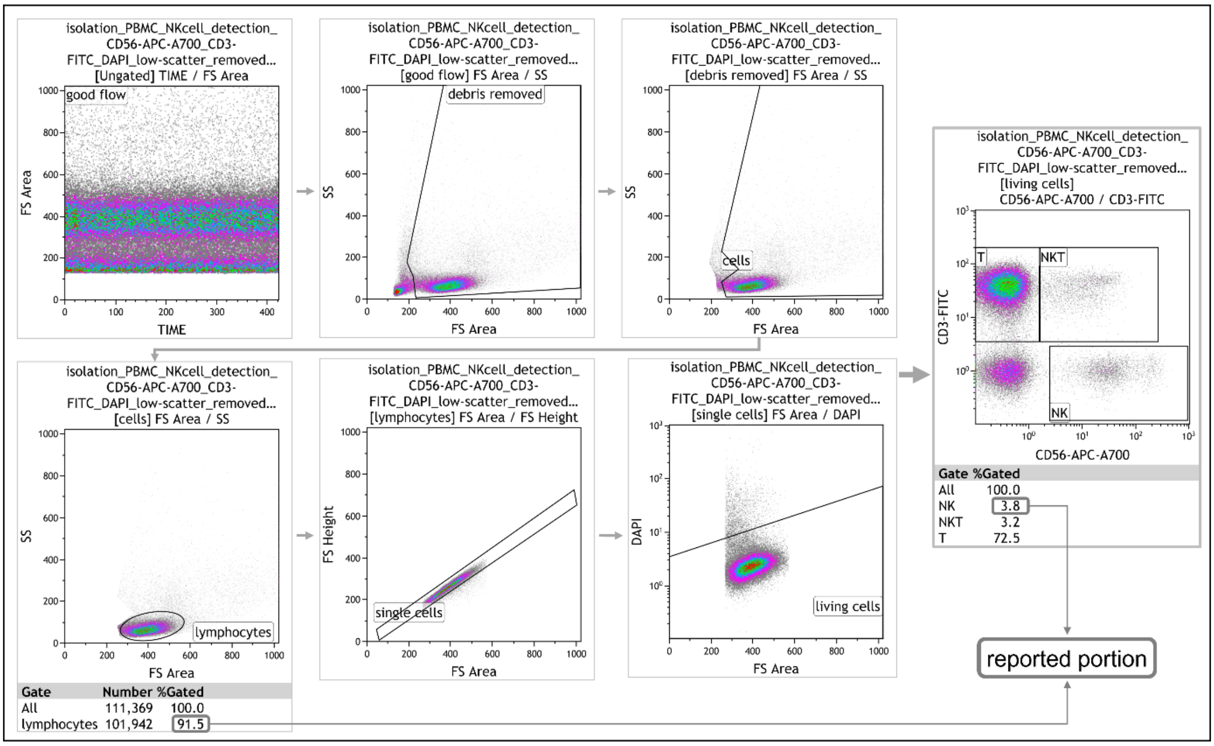

Figure S1: Representation of the gate settings during flow cytometry analysis to identify NK, NKT, and T cells. The gates were set to selectively contain events in a stable flow. Debris was removed by applying two scatter plots. A further gate was set to only contain lymphocytes. Doublets were removed through the gate single cells and dead cells were excluded by setting the gates to exclude DAPI stained cells. The remaining events were then visualized in a plot plotting CD56 against CD3 to allow identification of NK, NKT, and T cells.

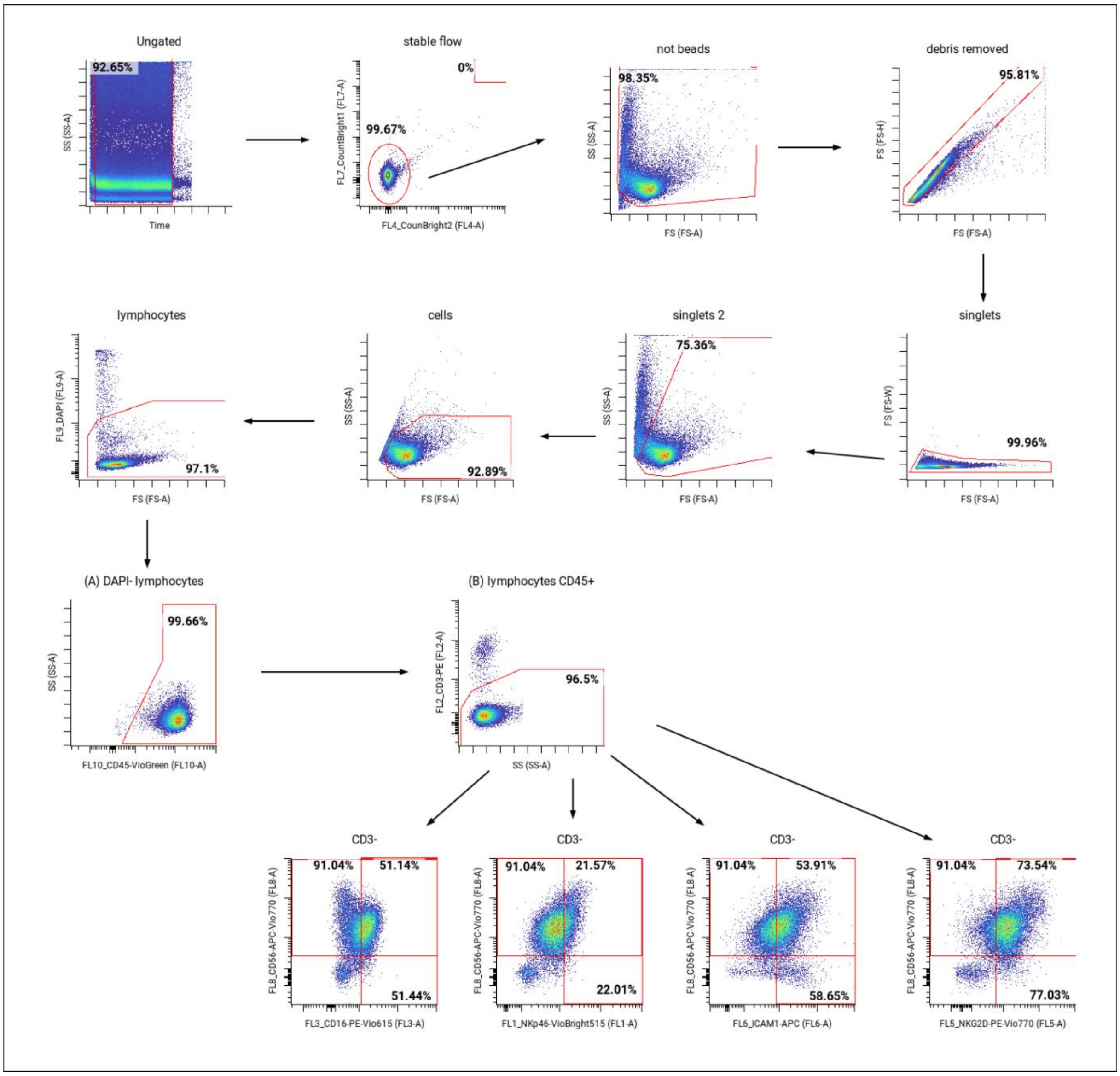

Figure S2. Representation of the gate settings during flow cytometry analysis to identify NK cells and characterize them regarding CD16, NKp46, ICAM-1, and NKG2D expression. The gates were set to selectively contain events in a stable flow. Although not applied, we designed the panel and analysis workflow with the option to incorporate counting beads for cell enumeration. Therefore, the strategy incorporates gates to differentiate beads from other events. Debris was removed and singlets were selected before cells and subsequently lymphocytes were selected, all based on scatter signals. A further gate was set to only contain living lymphocytes by discarding DAPI positive cells. Hematopoietic cell identity was verified by selecting CD45 positive cells. To exclude T and NKT cells CD3 negative cells were selected and illustrated on CD56 versus CD16, NKp46, ICAM-1, and NKG2D.

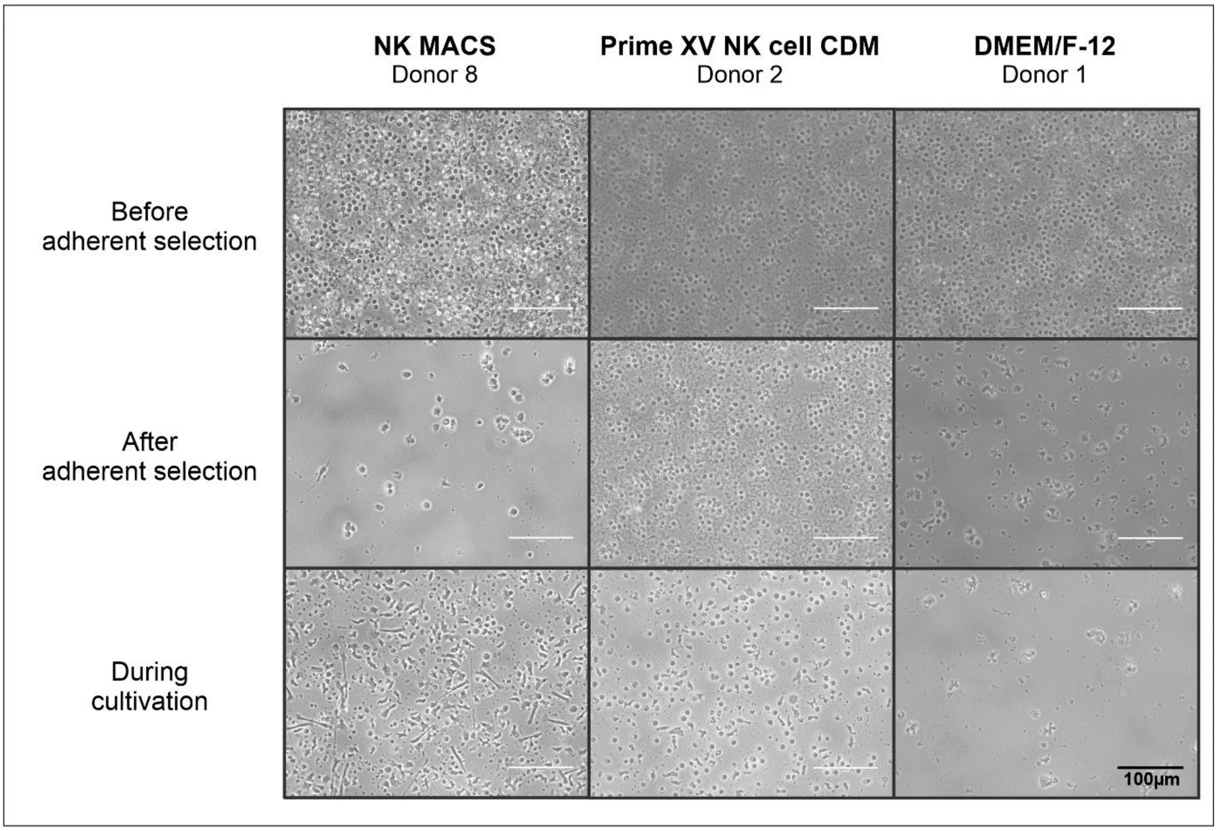

Figure S3. Microscopic images of freshly isolated, cultured, and adherently selected PBMCs in different media for NK cell enrichment. Overnight activated PBMCs before adherent selection, after adherent selection, and during cultivation. The scale (100 µm) shown on the bottom right applies to all images. For each medium, the images of one donor are shown representatively.

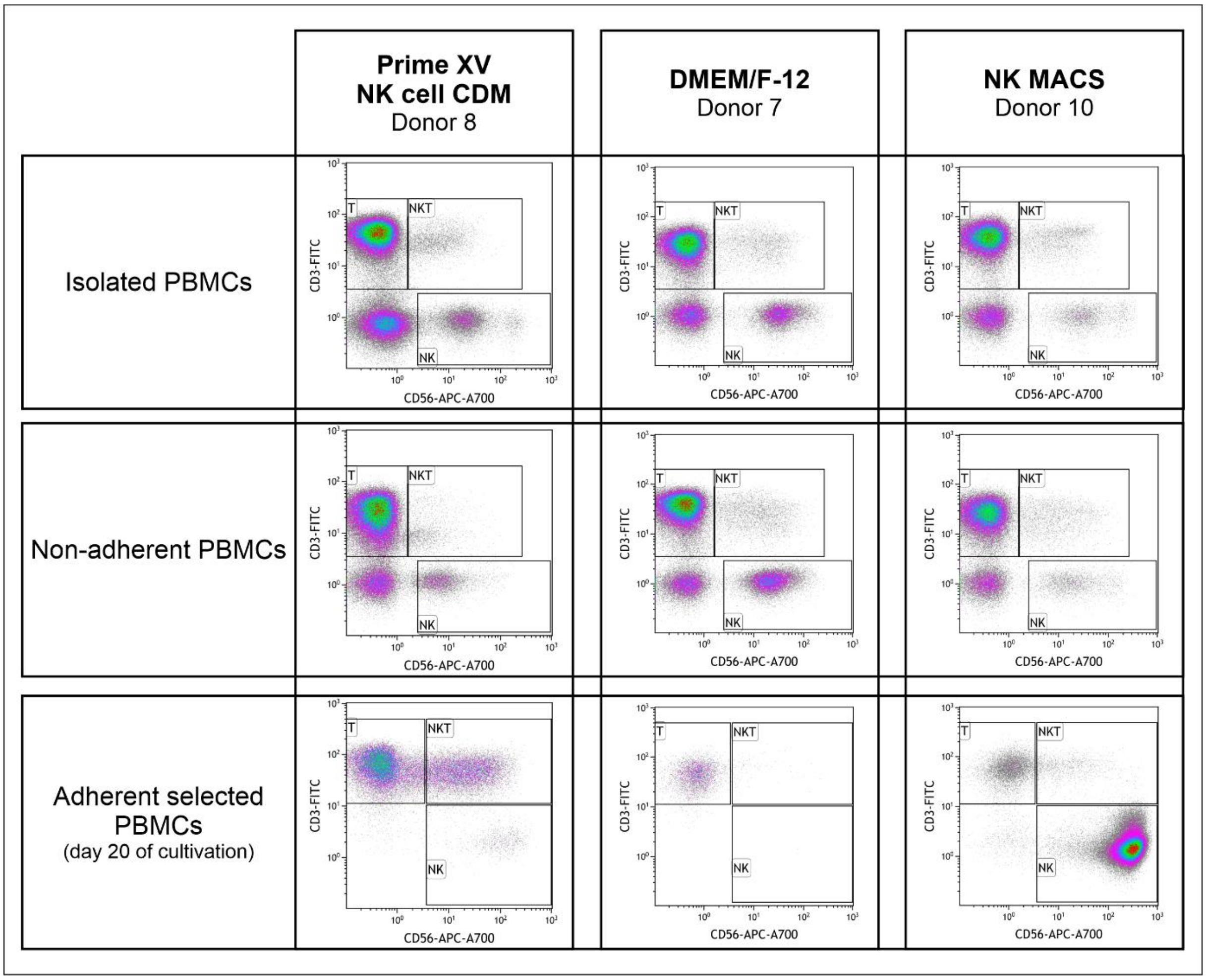

Figure S4. Flow cytometric analysis of freshly isolated, cultured, and adherently selected PBMCs in different media for NK cell enrichment. Freshly isolated PBMCs, removed non-adherent PBMCs and cultivated adherently selected PBMCs in PRIME-XV NK Cell CDM, DMEM/F-12 or NK MACS®. For each medium, the plots of one donor are shown representatively, the specific values measured are detailed in Table S1.

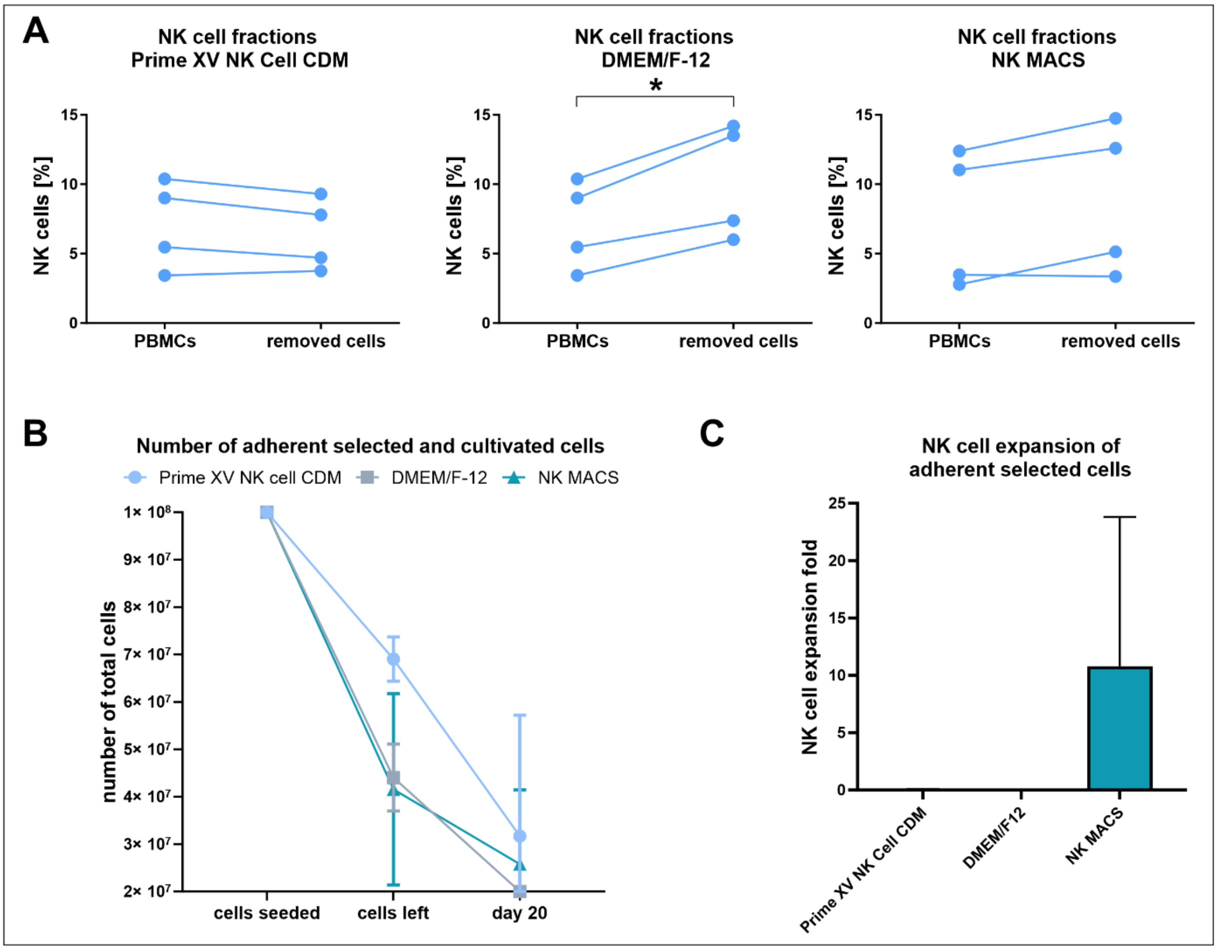

Figure S5. Characterization of the adherent selection method regarding removed cell fractions, total cell numbers and mean NK cell expansion after culture. Freshly isolated PBMCs were cultured in different media (PRIME-XV NK Cell CDM, DMEM/F-12 or NK MACS®) and adherently selected for NK cell enrichment (n = 4; mean ± SEM). **A:** Comparison of the NK cell percentages among seeded PBMCs and removed cells during adherent selection. The statistical analysis was performed per paired t-test (*p< 0.0332). **B:** Total number of PBMCs left after the adherent selection process. **C:** NK cell expansion fold of adherent selected cells, based on the NK cells left after the selection process (n = 4; mean ± SEM; SEM calculated via error propagation according to Gauss).

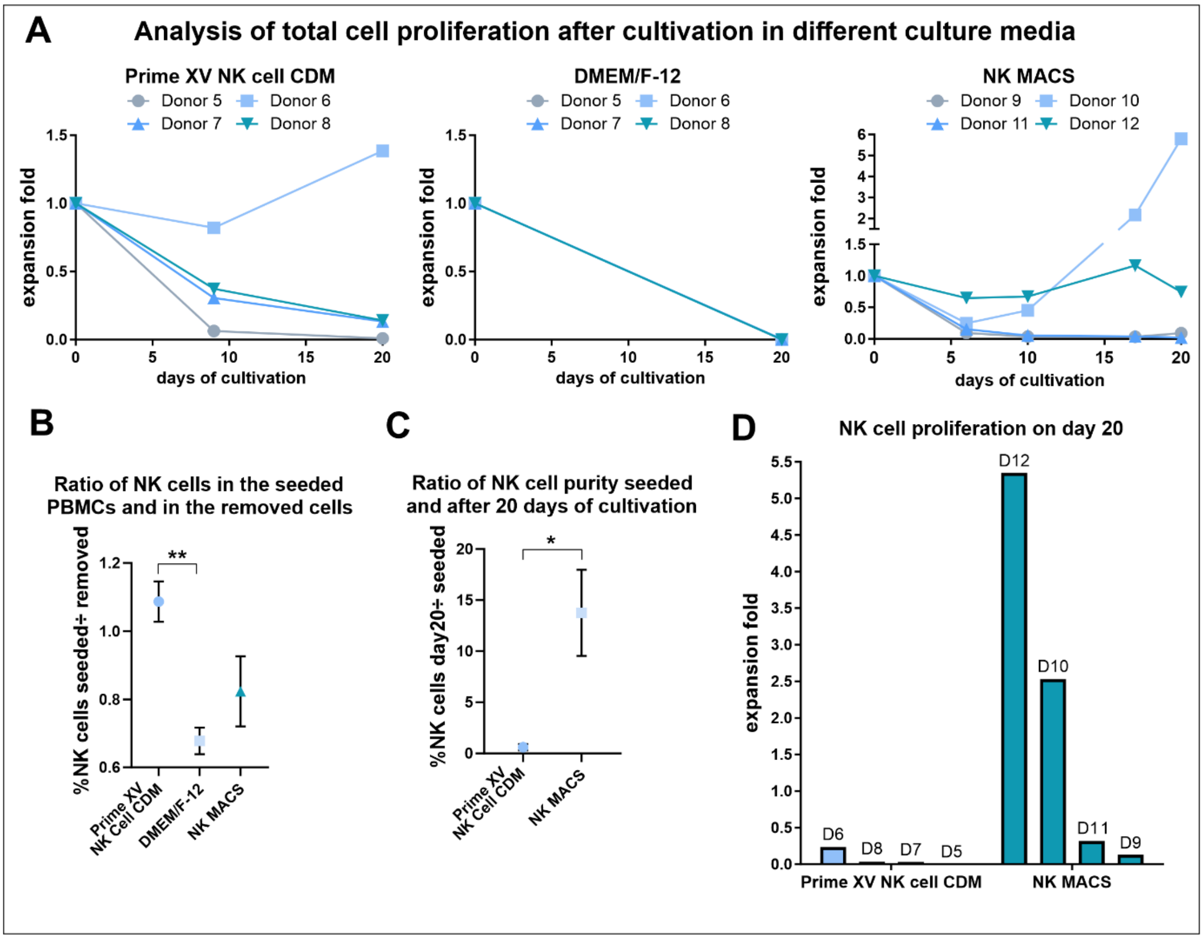

Figure S6. Characterization of the adherent selection method regarding individual donor expansion, removed NK cell fractions, and donor specific NK cell expansion after culture. Freshly isolated PBMCs were cultured in different media (PRIME-XV NK Cell CDM, DMEM/F-12 or NK MACS®) and adherently selected for NK cell enrichment. **A:** Expansion of adherently selected PBMCs during the cultivation period in different cell culture media (**p< 0.0021). The statistical analysis was performed per one-way ANOVA and applying Tukey correction for multiple comparisons. **C:** Ratio of the NK cell purity in the seeded PBMCs and in the adherently selected PBMCs after 20 days of cultivation (*p< 0.0332). The statistical analysis was performed per unpaired t-test. **B:** Ratio of NK cells in the seeded PBMCs and in the removed cells during the adherence-based selection (**p< 0.0021). The statistical analysis was performed per one-way ANOVA and applying Tukey correction for multiple comparisons. **C:** Ratio of the NK cell purity in the seeded PBMCs and in the adherently selected PBMCs after 20 days of cultivation (*p< 0.0332). The statistical analysis was performed per unpaired t-test. **C:** NK cell expansion of adherently selected PBMCs after three weeks of culture based on the NK cells seeded before the selection process for cells cultured in PRIME-XV NK Cell CDM or NK MACS® shown for each donor.

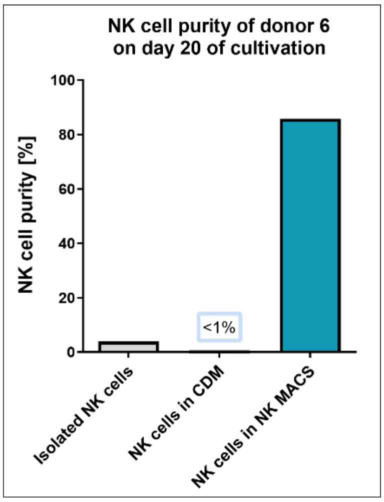

Figure S7. NK cell purity of adherent selected cells obtained from donor 6 cultured in different media on day 20 of cultivation. PBMCs were isolated and the NK cell purity was asset via flow cytometry. The adherence-based selection approach involved using PRIME-XV NK Cell CDM with fresh cells, while cryopreserved cells from this donor were utilized in the approach involving NK MACS® medium.

Table S1. Summary of the adherent selection experiment, including the median of the NK cell purity as well as T cell contamination. In addition, the number of NK cells in the isolated PBMCs, as well as enriched and expanded cells on day 20 of cultivation is given.

**Medium**	**Donor**	**NK cells [%]**	**T + NKT cells [%]**	**Number of NK cells [×10^6]**
Isolated PBMCs				
PRIME-XV NK Cell CDM (*n* = 4)	5	10.38%	47.00%	10.38
	6	3.43%	71.81%	3.43
	7	9.01%	46.75%	9.01
	8	5.47%	48.27%	5.47
DMEM/F-12 (*n* = 4)	5	10.38%	47.00%	10.38
	6	3.43%	71.81%	3.43
	7	9.01%	46.75%	9.01
	8	5.47%	48.27%	5.47
NK MACS® (*n* = 4)	9	2.78%	80.94%	2.78
	10	12.39%	48.70%	12.39
	11	3.48%	69.27%	3.48
	12	11.03%	54.74%	11.03
Adherently selected and expanded cells on day 20 of cultivation				
PRIME-XV NK Cell CDM (*n* = 4)	5	15.57%	23.77%	0.09
	6	0.74%	91.03%	0.8
	7	2.84%	81.71%	0.28
	8	2.23%	85.17%	0.18
DMEM/F-12 (*n* = 2)	5	2.28%	1.14%	>0.01
	7	0%	65.76%	0
**NK MACS®** (*n* = 4)	9	**39.25%**	55.71%	0.36
	10	**90.21%**	6.49%	31.35
	11	**88.87%**	7.25%	1.11
	12	**89.09%**	6.62%	59.01

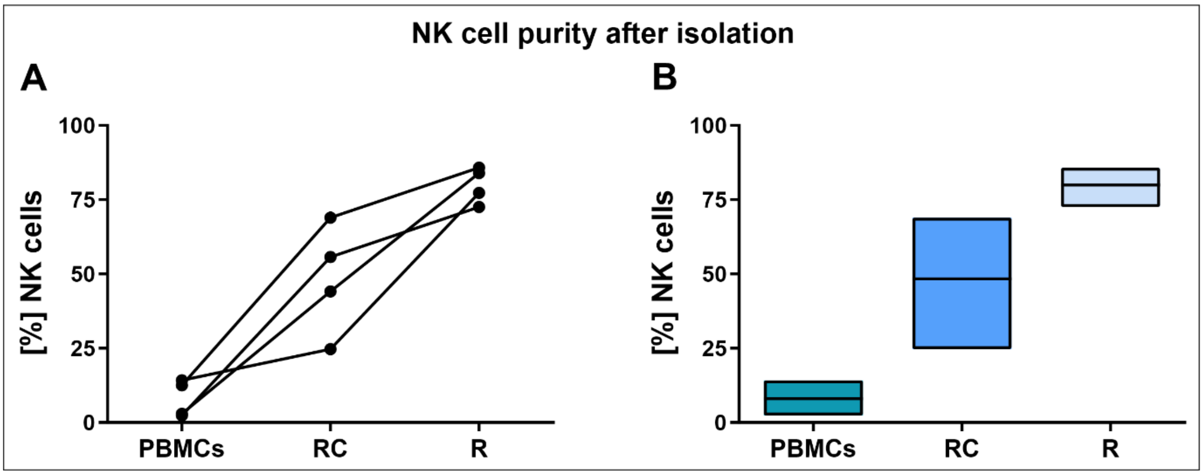

Figure S8. NK cell purity after isolation of NK cells using the RosetteSep™ human NK cell enrichment kit and PBMCs. For the isolation of NK cells, untreated (R) as well as two-fold concentrated (RC) buffy coats were used. **A:** NK cell purities of the respective donors regarding the different isolation strategies (*n* = 4). **B:** NK cell purities of the different isolation strategies (*n* = 4; mean ± SD).

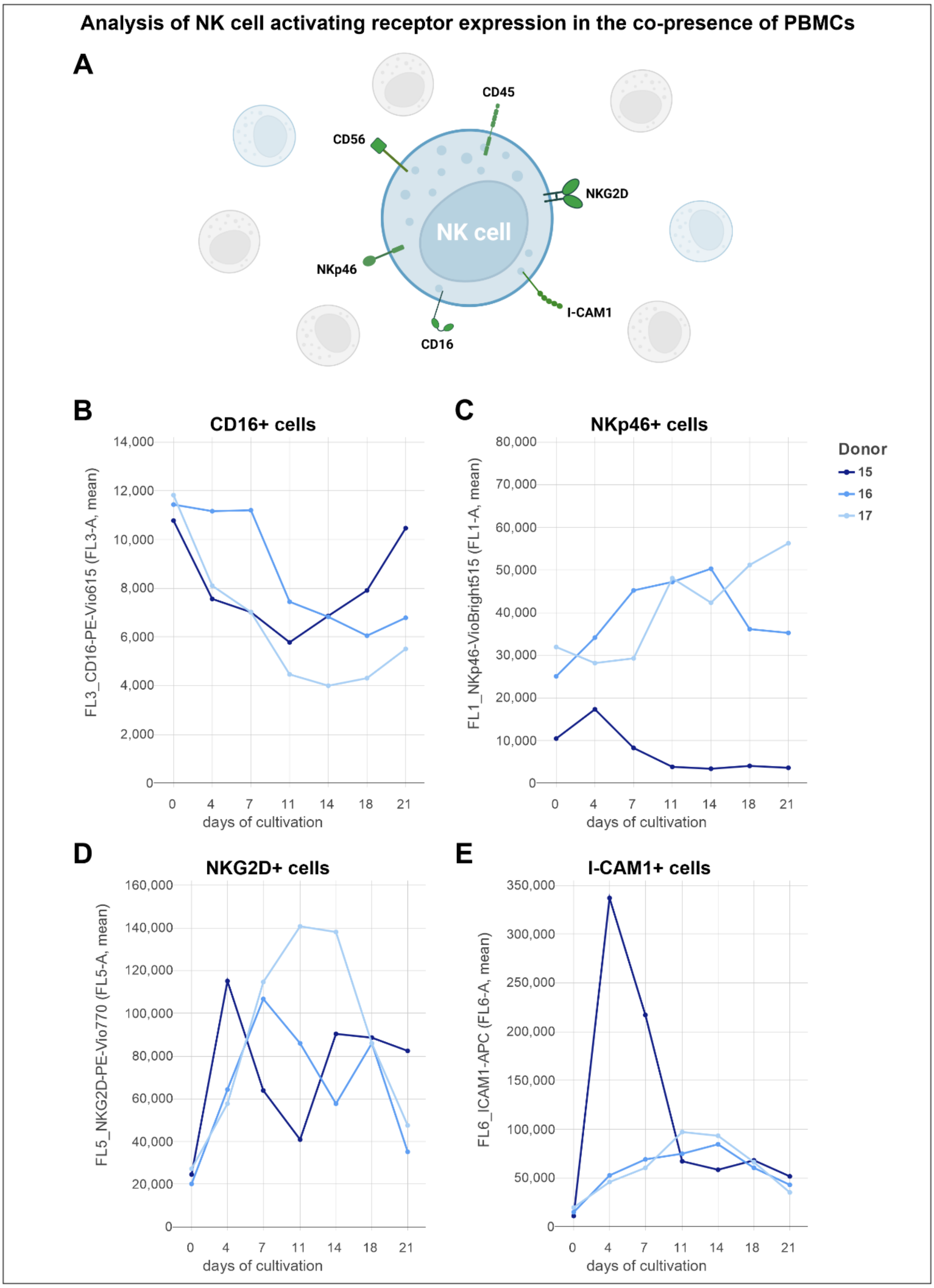

Figure S9. Mean expression levels of activating NK cell receptors on NK cells co-cultured PBMCs. Freshly isolated PBMCs were cultured in NK MACS® medium supplemented with human AB serum and 500 U/ml IL-2 for 21 days (n = 3). During the cultivation periods, the receptor expression of specific NK cell activation markers was determined through flow cytometric analysis. **A:** Schematic representation of the expression of NK cell activating receptors in the co-presence of other PBMCs. **B:** Mean fluorescence intensity of the NK cell activation marker CD16. **C:** Mean fluorescence intensity of the NK cell activation marker NKp46. **D:** Mean fluorescence intensity of the NK cell activation marker NKG2D. **E:** Mean fluorescence intensity of the NK cell activation marker I-CAM1.

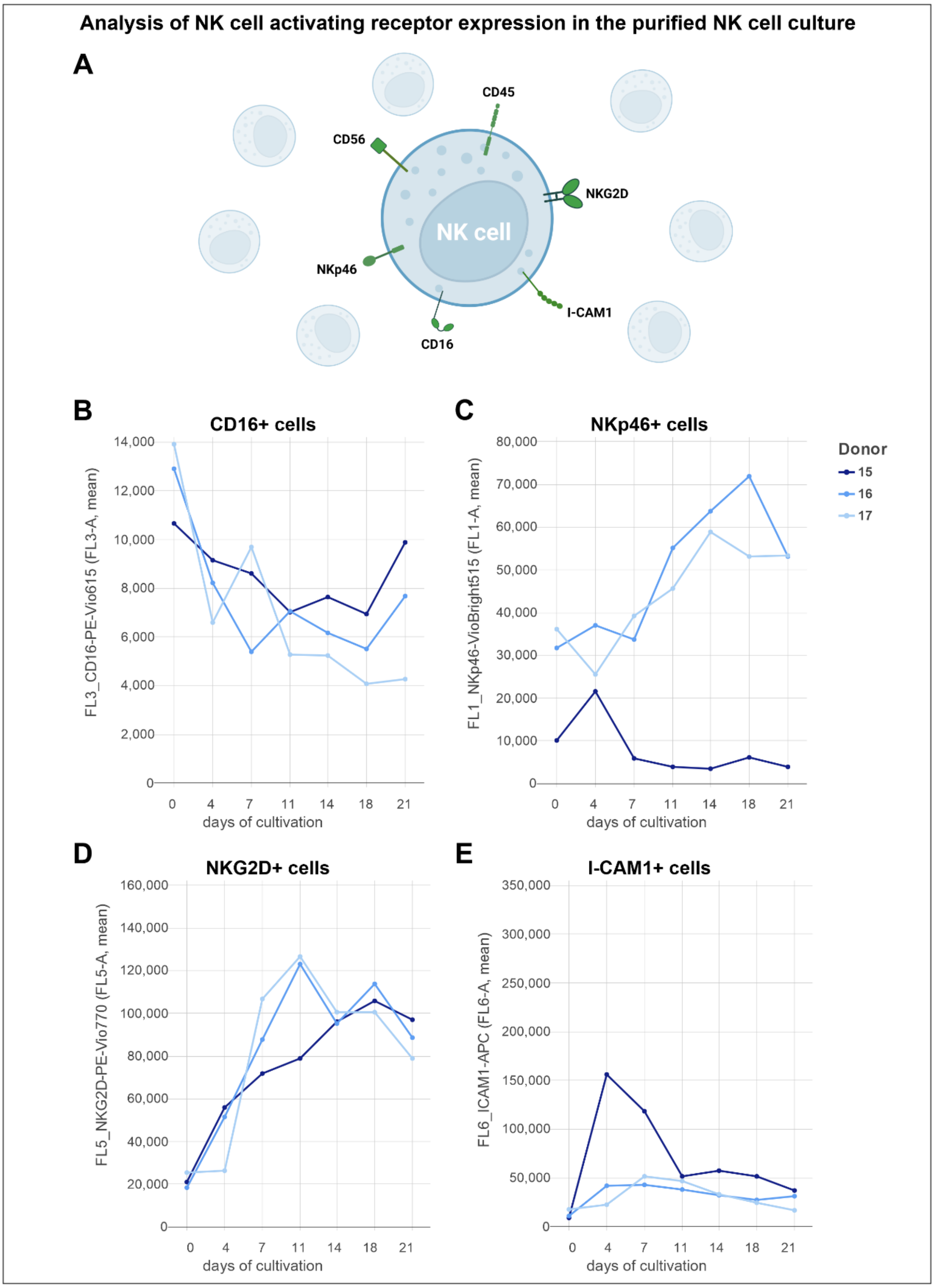

Figure S10. Mean expression levels of activating NK cell receptors on NK cells cultured without PBMCs. Freshly isolated and purified NK cells were cultured in NK MACS® medium supplemented with human AB serum and 500 U/ml IL-2 for 21 days ($n = 3$). During the cultivation periods, the receptor expression of specific NK cell activation markers was determined through flow cytometric analysis. **A:** Schematic representation of the expression of NK cell activating receptors in the absence of other PBMCs. **B:** Mean fluorescence intensity of the NK cell activation marker CD16. **C:** Mean fluorescence intensity of the NK cell activation marker NKp46. **D:** Mean fluorescence intensity of the NK cell activation marker NKG2D. **E:** Mean fluorescence intensity of the NK cell activation marker I-CAM1.
